# Acyl-chain saturation regulates the order of phosphatidylinositol 4,5-bisphosphate nanodomains

**DOI:** 10.1038/s42004-021-00603-1

**Published:** 2021-11-29

**Authors:** Luís Borges-Araújo, Marco M. Domingues, Alexander Fedorov, Nuno C. Santos, Manuel N. Melo, Fábio Fernandes

**Affiliations:** 1 Institute for Bioengineering and Biosciences (IBB) and Associate Laboratory i4HB-Institute for Health and Bioeconomy, Instituto Superior Técnico, Universidade de Lisboa, Lisbon, Portugal; 2 Instituto de Tecnologia Química e Biológica António Xavier, Universidade Nova de Lisboa, Av. da República, 2780-157 Oeiras, Portugal; 3 Instituto de Medicina Molecular, Faculdade de Medicina, Universidade de Lisboa, Av. Prof. Egas Moniz, 1649-028 Lisbon, Portugal; 4 Department of Bioengineering, Instituto Superior Técnico, Universidade de Lisboa, 1049-001 Lisbon, Portugal

**Keywords:** Phospholipids, Biophysical chemistry

## Abstract

Phosphatidylinositol 4,5-bisphosphate (PI(4,5)P_2_) plays a critical role in the regulation of various plasma membrane processes and signaling pathways in eukaryotes. A significant amount of cellular resources are spent on maintaining the dominant 1-stearoyl-2-arachidonyl PI(4,5)P_2_ acyl-chain composition, while less abundant and more saturated species become more prevalent in response to specific stimuli, stress or aging. Here, we report the impact of acyl-chain structure on the biophysical properties of cation-induced PI(4,5)P_2_ nanodomains. PI(4,5)P_2_ species with increasing levels of acyl-chain saturation cluster in progressively more ordered nanodomains, culminating in the formation of gel-like nanodomains for fully saturated species. The formation of these gel-like domains was largely abrogated in the presence of 1-stearoyl-2-arachidonyl PI(4,5)P_2._ This is, to the best of our knowledge, the first report of the impact of PI(4,5)P_2_ acyl-chain composition on cation-dependent nanodomain ordering, and provides important clues to the motives behind the enrichment of PI(4,5)P_2_ with polyunsaturated acyl-chains. We also show how Ca^2+^-induced PI(4,5)P_2_ nanodomains are able to generate local negative curvature, a phenomenon likely to play a role in membrane remodeling events.

## Abstract (web summary)

Phosphatidylinositol-4,5-bisphosphates are involved in membrane regulation and signalling in eukaryotes, with the distribution of acyl chains changing in response to stimuli. Here, molecular dynamics simulations and fluorescence spectroscopy experiments show that increasing acyl chain saturation increases the ordering of lipid nanodomains in artificial membranes.

## Introduction

Phosphoinositides are an important class of glycerophospholipids, which play a variety of diverse and specific roles across several eukaryotic membrane structures. Phosphatidylinositol 4,5-bisphosphate (PI(4,5)P_2_) is one of the most abundant phosphoinositides, comprising ~1% of the total membrane phospholipids in mammalian cells^1^. It is mostly localized in the inner leaflet of the plasma membrane, where it plays a crucial role in multiple pathways, especially those related with membrane dynamics^2^. PI(4,5)P_2_ has been associated with several membrane processes, such as vesicle trafficking^3,4^, cytoskeletal regulations^5^, ion channel function^6^, viral assembly^7^, and budding^8^.

It is remarkable that a single lipid class present at low and constant steady-state levels can act as an important regulator in so many different, yet simultaneous, signaling pathways^9^. Lateral organization of PI(4,5)P_2_ is critical to that end. It is thought that PI(4,5)P_2_ is heterogeneously dispersed across the membrane, in localized PI(4,5)P_2_-enriched clusters at particular sites and timings^10^. These clusters are promoted not only through localized depletion and synthesis of PI(4,5)P_2_ but also by the formation of PI(4,5)P_2_ nanodomains, promoted by the interaction of its negatively charged phosphorylated headgroup with divalent cations (such as Ca^2+^ and Mg^2+^) or positively charged proteins^11,12^. It has been shown that divalent cations have the ability to induce the dramatic segregation of PI(4,5)P_2_ to form highly enriched nanodomains, even at physiological concentrations of both lipid and cation^10,13^. Interactions between the anionic headgroup phosphates and divalent cations not only screen the electrostatic repulsion between headgroups but can also crosslink adjacent lipids. These PI(4,5)P_2_ clusters have been shown to have higher affinity for more ordered lipid phases than the monomeric species^14^.

The higher affinity of clustered PI(4,5)P_2_ towards ordered membrane domains is puzzling, given its bulky headgroup and acyl-chain unsaturation profile, which are expected to strongly favor incorporation into more disordered membrane phases. Although most phospholipids show considerable acyl-chain composition diversity, PI(4,5)P_2_ molecules are highly enriched in specific acyl chains^15^. In fact, the most frequent fatty acyl-chain pair for PI(4,5)P_2_ in mammalian cells is 1-stearoyl-2-arachidonyl (18:0 20:4)^16^. This combination consists of up to 70% of the total PI(4,5)P_2_ pool in some cases, particularly in brain tissue^17–19^. This enrichment is likely the combined outcome of specific substrate specificity for 1-stearoyl-2-arachidonyl-glycerol in several enzymes in the phosphatidylinositol cycle and of phosphoinositide acyl-chain remodeling via the Land’s cycle^17^.

The biological functions that call for this specific enrichment are still not clear. It has been shown that arachidonate (20:4) and other polyunsaturated fatty acids such as docosahexaenoate (22:6), when at the *sn*-2 position, facilitate membrane shaping and fission activities^20^. In addition, asymmetric *sn*-1-saturated-*sn*-2-polyunsaturated phospholipids have been shown to promote efficient membrane vesiculation, while maintaining low membrane permeability^20^. PI(4,5)P_2_ has been associated with several stages of both endocytosis and exocytosis, being considered an important mediator of synaptic vesicle trafficking, where this composition might provide mechanical benefits^3^. Despite the dominant presence of the canonical 18:0 20:4 species, PI(4,5)P_2_ acyl-chain composition is not fully uniform and includes a larger range of acyl-chain lengths and unsaturation profiles^19^. PI(4,5)P_2_ molecular species exhibiting no polyunsaturation or even fully saturated acyl chains are less abundant, but have been reported to become more prevalent in response to certain stimuli^21^, stress^19,21^, aging^19^, or in cancer^22^. In one of these cases, the levels of PI(4,5)P_2_ with this profile of acyl-chain composition even surpassed the canonical 18:0 20:4 composition^21^.

However, there is still hardly any research done on the impact of these more saturated species in PI(4,5)P_2_ organization. More saturated acyl chains are expected to lead to a more ordered and less fluid membrane landscape. PI(4,5)P_2_ species exhibiting lower unsaturation or even full saturation are likely to present significantly different lateral distribution that that of the canonical 18:0 20:4 form. Considering the importance of PI(4,5)P_2_ for sorting of membrane proteins, such difference is likely to have profound influence in many PI(4,5)P_2_-dependent cellular processes. In addition, interactions of proteins with specific PI(4,5)P_2_ acyl chains have already been reported. HIV-1 Gag polyprotein was found to sequester unsaturated acyl chains from PI(4,5)P_2_ and store them in a hydrophobic pocket during the membrane-anchoring process^23^. Several enzymes, such as phosphatidylinositol-4-phosphate-5-kinase, also show preference for some acyl-chain configurations when using the lipid as either a substrate or as an activator^17^. Further insight on how the acyl-chain profile can influence PI(4,5)P_2_ biophysical properties may help in shedding some light on how it can affect downstream PI(4,5)P_2_-dependent processes.

In this study, we investigated three different PI(4,5)P_2_ acyl-chain configurations, representative of the broad spectrum observed in vivo, in order to better understand the extent of the impact of acyl-chain saturation on PI(4,5)P_2_ biophysical properties and on cation-induced PI(4,5)P_2_ nanodomains. Through a combination of atomic force microscopy (AFM), coarse-grained (CG) molecular dynamics (MD) simulations and fluorescence spectroscopy techniques, we show that different PI(4,5)P_2_ acyl-chain compositions lead to the formation of Ca^2+^-induced nanodomains with distinct biophysical properties. Specifically, we observed that as saturation increases, PI(4,5)P_2_ forms more ordered nanodomains, which can ultimately culminate in the formation of gel-like nanodomains with the fully saturated acyl-chain composition. These results provide an important biophysical insight on the motives behind the conserved pattern of enrichment of PI(4,5)P_2_ with polyunsaturated acyl chains.

PI(4,5)P_2_ is known to directly regulate several membrane-remodeling events^3,4,24,25^. Although PI(4,5)P_2_ is essential for membrane fusion processes, such as soluble NSF attachment receptor (SNARE)-dependent membrane fusion, it has an intrinsic positive curvature, which is expected to restrain the formation of the negatively curved intermediates necessary for some of these mechanisms^3,4,24,25^. Using CG MD simulations, we also show here how PI(4,5)P_2_ nanodomains associate with negatively curved membranes. Generation of negative curvature by clustered PI(4,5)P_2_ is likely to play a major rule in membrane-remodeling events.

## Results

### Acyl-chain composition does not influence the formation of PI(4,5)P_2_ nanodomains

In order to study the influence of acyl-chain composition on the properties of PI(4,5)P_2_ nanodomains, three compositions were chosen as representative of the broad spectrum seen in vivo. (18:0 20:4) PI(4,5)P_2_ was an obvious choice, as it is the dominant species detected in mammalian cells. To represent the less abundant, more saturated species, (18:1)_2_ and (16:0)_2_ PI(4,5)P_2_ were chosen.

To characterize the lateral distribution of PI(4,5)P_2_ in lipid membranes, both in the presence and in the absence of divalent cations, we employed the fluorescent analog TF-PI(4,5)P_2_. This analog has already been successfully used in the detection of PI(4,5)P_2_ nanodomains in large unilamellar vesicles (LUVs)^10,13^. TF-PI(4,5)P_2_ is capable of undergoing homo Förster resonance energy transfer (FRET) and, as a result, fluorescence depolarization takes place upon enrichment of the analog within nanoclusters in the membrane. Thus, nanoclustering of PI(4,5)P_2_ can be followed through a decrease in fluorescence anisotropy (<*r*>) of TF-PI(4,5)P_2_^10^. During Ca^2+^-induced clustering, the presence of higher concentrations of unlabeled PI(4,5)P_2_ contributes to the formation of larger clusters, promoting TF-PI(4,5)P_2_ sequestration, which is detectable through a decrease of <*r*>^10^.

**Fig. 1:**
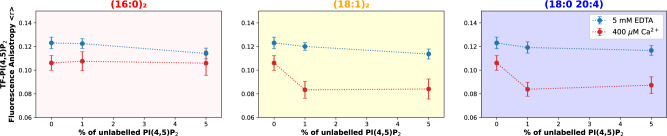
Cation-induced clusters are formed independently of PI(4,5)P_2_ acyl-chain composition as seen by homo-FRET of the TF-PI(4,5)P_2_ analog. PI(4,5)P_2_ cluster formation was determined through the incorporation of 0.1 mol% of TF-PI(4,5)P_2_ in LUVs containing POPC and increasing concentrations of unlabeled PI(4,5)P_2_. The experiments were carried out for the three unlabeled PI(4,5)P_2_ species, with different acyl-chain composition. TF-PI(4,5)P_2_ fluorescence anisotropy (*<r>*) values were measured in the presence (400 µM Ca^2+^, red) and absence (5 mM EDTA, blue) of calcium. Error bars represent the SD from *N* = 3 independent experiments. A significant decrease in TF-PI(4,5)P_2_ fluorescence anisotropy in the presence of calcium is observed for the three PI(4,5)P_2_ species ((16:0)2 PI(4,5)P_2_: F(1,12) = 17.87, *p* = 0.0012; (18:1)_2_ PI(4,5)P_2_: F(1,12) = 96.84; *p* < 0.0001; (18:0 20:4) PI(4,5)P_2_: F(1,12) = 107.7, *p* < 0.0001).

TF-PI(4,5)P_2_ was incorporated in 1-palmitoyl-2-oleoyl-glycero-3-phosphocholine (POPC):PI(4,5)P_2_ LUVs at an analog to lipid ratio of 0.1 mol%. At such low concentrations, only residual FRET takes place in the absence of clustering^10^. As expected, we observed for all compositions that, in the absence of free calcium, the recovered fluorescence anisotropy of the analog, <*r*>, was 0.125 (Fig. 1), a value consistent with monodispersed TF-PI(4,5)P_2_ with no aggregation^10^.

A concentration of 400 μM Ca^2+^ was used for Ca^2+^-induced PI(4,5)P_2_ clustering experiments. This concentration is well within the expected levels of the divalent cation on local Ca^2+^ nanodomains after opening of channels^26^. Importantly, in the presence of 400 μM Ca^2+^, the fluorescence anisotropy decreased for all compositions, which is indicative of higher FRET and sequestration of the analog into PI(4,5)P_2_ clusters, confirming that aggregation occurs independently of acyl-chain composition. Increasing concentrations of unlabeled PI(4,5)P_2_ were used to characterize the concentration dependence of clustering. For the (18:1)_2_ and (18:0 20:4) PI(4,5)P_2_ species in the presence of calcium, a similar decrease in anisotropy with increasing concentrations of unlabeled PI(4,5)P_2_ is observed (F(1,12) = 0.1445, *p* = 0.7105). These results suggest that the extent of PI(4,5)P_2_ clustering obtained for (18:1)_2_ and (18:0 20:4) is comparable. Surprisingly, the decrease in *<r*> for (16:0)_2_ PI(4,5)P_2_ in the presence of calcium is independent from the concentration of unlabeled PI(4,5)P_2_ (F(2,12) = 1.023, *p* = 0.3887).

Fluorescence lifetime ($\overline \tau$) of the analog is also decreased upon insertion into PI(4,5)P_2_ clusters^10^. In the absence of calcium, $\overline \tau$ is identical for all PI(4,5)P_2_ species (Supplemantary Fig. 1), reflecting the comparable local environment provided by the bulk membrane, composed mostly of POPC. When 400 μM Ca^2+^ was included, the fluorescence lifetime of the analog decreased similarly for all PI(4,5)P_2_ species (Supplementary Fig. 1). The data for fully saturated PI(4,5)P_2_ were once again puzzling, as the lifetime of the analog greatly increased when higher concentrations of (16:0)_2_ PI(4,5)P_2_ were used. Incorporation of the analog into PI(4,5)P_2_ nanodomains is confirmed by fluorescence correlation spectroscopy (FCS) measurements (Supplementary Fig. 2), which show a decrease in the TF-PI(4,5)P_2_ diffusion coefficient across the three acyl-chain compositions consistent with the sequestration into Ca^2+^-induced clusters. Previous results from our laboratory have already shown that although Ca^2+^ is able to induce a minor decrease in diffusion coefficient of membranes enriched in other anionic lipids, the impact on PI(4,5)P_2_-containing membranes is considerably larger, reflecting the specificity of the process^10^. It is noteworthy that the diffusion coefficient measured here reflects the diffusion of the cluster and the diffusion rates inside clusters fall quite below the resolution of the confocal microscope and are not resolved by FCS.

The interpretation of these results is simple for the mono- and polyunsaturated compositions, and clearly indicates the sequestration of the analog within PI(4,5)P_2_ nanodomains of similar properties. However, the photophysical behavior of the analog in the presence of Ca^2+^-induced (16:0)_2_ PI(4,5)P_2_ clusters suggests that the properties of these nanodomains differ dramatically from those obtained with unsaturated species. Given the saturated acyl chains of (16:0)_2_ PI(4,5)P_2_, it is possible that clustering of this lipid gives rise to highly ordered domains, whereas unsaturated PI(4,5)P_2_ species give rise to more fluid nanodomains. This would be consistent with the fluorescence anisotropy and lifetime of the analog within (16:0)_2_ PI(4,5)P_2_ domains. In fact, fluorescence anisotropy values of membrane probes in ordered membrane phases are known to increase due to limited rotational diffusion of the fluorophore, while the fluorescence lifetime is often increased^27^.

**Fig. 2:**
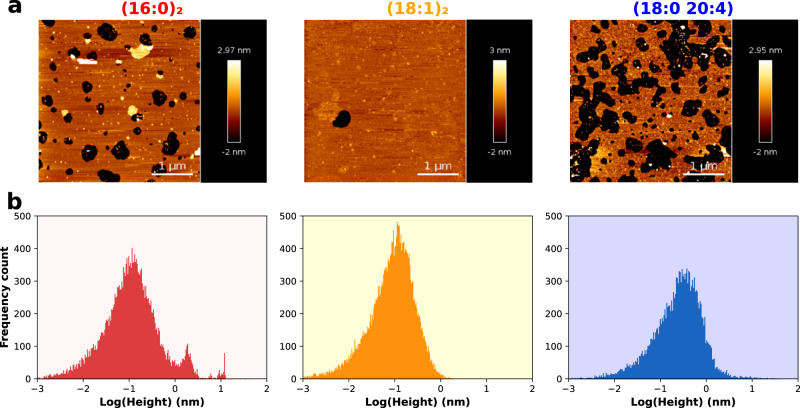
Cation-induced clusters are formed independently of PI(4,5)P_2_ acyl-chain composition, as seen by AFM measurements of supported lipid bilayers. PI(4,5)P_2_ cluster formation was detected through the AFM measurement of SLBs containing DOPC and 5% PI(4,5)P_2_. Experiments were carried out for the three PI(4,5)P_2_ species in study and figures are labeled according to the acyl-chain composition of the PI(4,5)P_2_ species employed. Topographical images were acquired (**a**) and analyzed with first- or second-level flattening, using the JPK data processing software, from which the membrane heigh profiles (**b**) were obtained. Dark patches correspond to defects in the supported membrane. The height values of each pixel (higher than 0 nm) were transformed into log_10_ value, avoiding the influence of the height values of defects on the histograms. The frequency count axis represents the number of data points grouped into each bin on the height histograms.

The nature of the different Ca^2+^-induced PI(4,5)P_2_ clusters was also analyzed by AFM on supported lipid bilayers (SLBs) of 1,2-dioleoyl-sn-glycero-3-phosphocholine (DOPC):PI(4,5)P_2_ 95:5 (Fig. 2a). DOPC was used instead of POPC, as was done for all other experiments, as it can undergo lipid interdigitation and enhance contrast between the bulk membrane and other phases, in this case, PI(4,5)P_2_ nanodomains. All three PI(4,5)P_2_ species are confirmed to produce nanodomains. The properties of (16:0)_2_ PI(4,5)P_2_ clusters were once more distinguishable from the other PI(4,5)P_2_ species: although the height of nanodomains formed from unsaturated PI(4,5)P_2_ is found to be only slightly above that of the bulk DOPC bilayer, the height of (16:0)_2_ PI(4,5)P_2_ domains was, on average, 2 nm thicker than the rest of the membrane. This difference gives rise to a clear second peak in the histograms of bilayer height (Fig. 2b). This large increase in bilayer thickness suggests a dramatic reorganization of lipid structure for (16:0)_2_ PI(4,5)P_2_. AFM studies of SLBs composed of DOPC and 1,2-diplamitoyl-sn-glycero-3-phosphocholine (DPPC) have shown DPPC gel domains to be roughly 2 nm thicker than the bulk membrane^28^, whereas Martini CG MD simulations of fluid DOPC:(16:0)_2_ PI(4,5)P_2_ systems have shown differences in lipid height of roughly 1 nm over a single lipid bilayer (Supplementary Fig. 9). In the context of (16:0)_2_ PI(4,5)P_2_ gel-like nanodomains, it is likely that this difference in height would be slightly larger, in the 2 nm range seen experimentally.

Although both (16:0)_2_ PI(4,5)P_2_ and (18:1)_2_ PI(4,5)P_2_ mostly gave rise to nanodomains of 30–50 nm diameter, the polyunsaturated PI(4,5)P_2_ promoted the formation of smaller clusters, predominantly below these values (Supplementary Fig. 3). The nanodomains observed for all PI(4,5)P_2_ species were often unstable in SLBs and substantial fractions were seen to cleave off from the mica surface during the washing steps where unfused vesicles are rinsed from the surface. This gave rise to the dark patches observed in Fig. 2a. Given the large headgroup of PI(4,5)P_2_, it is likely that the curvature of these nanodomains differs considerably from the bulk DOPC, which can justify the observed instability.

### Ca^2+^-induced clustering leads to an increase in local membrane order, even for polyunsaturated acyl-chain compositions

The fluorescent analog and AFM results strongly indicate dramatic differences in nanodomain order for the different PI(4,5)P_2_ species. To quantify these differences, we made use of 1-(4-Trimethylammoniumphenyl)-6-phenyl-1,3,5-hexatriene *p*-toluenesulfonate (TMA-DPH), a membrane probe commonly used to study membrane fluidity^29^. As in these experiments PI(4,5)P_2_ only comprises a small percentage of membrane lipids, measuring the average membrane order is bound to underestimate any change observed within the phosphoinositide-rich domains. The cationic trimethylammonium moiety of TMA-DPH should be anchored near the phosphate and carbonyl/ester region of the membrane^29^ to maximize favorable interactions with electronegative oxygen atoms. Electrostatic attraction is expected to cause TMA-DPH to prefer the vicinity of the anionic PI(4,5)P_2_ molecules and their nanodomains, thus providing a strategy to probe the PI(4,5)P_2_ local environment.

**Fig. 3:**
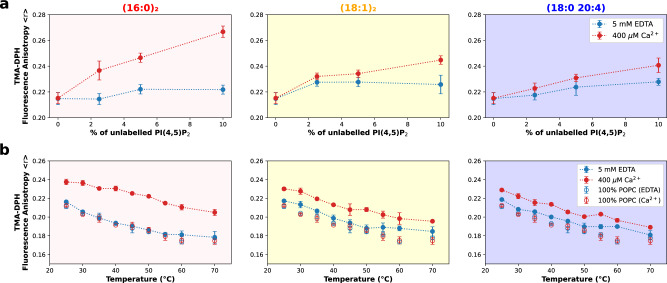
TMA-DPH fluorescence spectroscopy shows that calcium-induced PI(4,5)P_2_ nanodomains are more ordered than monodisperse PI(4,5)P_2_, even for unsaturated acyl-chain compositions. PI(4,5)P_2_ local membrane order was determined through the incorporation of TMA-DPH at a 1:300 lipid ratio in MLVs containing POPC and increasing concentrations of unlabeled PI(4,5)P_2_. The experiments were done for the three acyl-chain compositions in study. For all acyl-chain compositions analyzed here, an increase in local membrane order takes place upon Ca^2+^-induced PI(4,5)P2 nanodomain formation ((16:0)_2_ PI(4,5)P_2_: F(1,16) = 148.3, *p* = 0.0001; (18:1)_2_ PI(4,5)P_2_: F(1,16) = 19.71, *p* = 0.0004; (18:0 20:4) PI(4,5)P_2_: F(1,16) = 12.23, *p* < 0.0030). TMA-DPH fluorescence anisotropy (*<r>*) (**a**) was measured in the presence (400 µM Ca^2+^, red) and absence (5 mM EDTA, blue) of calcium. The TMA-DPH response to a temperature gradient (**b**) was measured in samples composed of POPC:PI(4,5)P_2_ 95:5. Pure POPC samples were measured as controls. All samples were measured both in the presence and absence of calcium. Error bars for all measurements represent the SD from *N* = 3 independent experiments.

The fluorescence anisotropy of TMA-DPH was measured in multilamellar vesicles (MLVs) composed of POPC and increasing concentrations of the different species of PI(4,5)P_2_ (Fig. 3a). Although in the absence of calcium, a minor increase in <*r*>_TMA-DPH_ is observed for higher concentrations of all PI(4,5)P_2_ variants, the increase is more evident in the presence of the divalent cation. A similar behavior is observed for the fluorescence lifetime of the probe (Supplementary Fig. 4). Surprisingly, these results show that for all acyl-chain compositions analyzed here, an increase in local membrane order takes place upon Ca^2+^-induced PI(4,5)P_2_ nanodomain formation. The extent of the increase in membrane order correlates with the degree of saturation in the acyl chains, with much higher increases in <*r*>_TMA-DPH_ for the fully saturated species and more moderate changes observed for the polyunsaturated PI(4,5)P_2_.

Once more, the behavior of (16:0)_2_ PI(4,5)P_2_ completely stands out from that of the other compositions, showing a much larger increase in *<r*>_TMA-DPH_ in the presence of calcium, whereas presenting a similar behavior in the absence of the divalent cation. As a comparison, a study on the impact of cholesterol on POPC membrane order found a similar increase in *<r*>_TMA-DPH_ only upon adding 40% of the sterol^30^.

In order to evaluate the stability of these structures, we measured *<r*>_TMA-DPH_ in liposomes containing 5% PI(4,5)P_2_ at increasing temperatures (Fig. 3b). In the absence of calcium, the behavior of all compositions was similar to that of pure POPC control membranes. The *<r*>_TMA-DPH_ decreases due to increased dynamics as the temperature increases. In the presence of calcium, *<r*>_TMA-DPH_ was noticeably higher throughout the range of tested temperatures and no clear inflection point is observed. These results confirm that, within this temperature range, there is no temperature dependence for the formation of PI(4,5)P_2_ nanodomains. Remarkably, unlike the solid-like gel phase domain of saturated lipids such as (16:0)_2_ PC (DPPC), which exhibit phase transition to fluid phase at a melting temperature of 41 °C^31^, the highly ordered phase created by (16:0)_2_ PI(4,5)P_2_ is stable up to 70 °C.

### Ca^2+^ induces the formation of (16:0)_2_ PI(4,5)P_2_ gel-like nanodomains

**Fig. 4:**
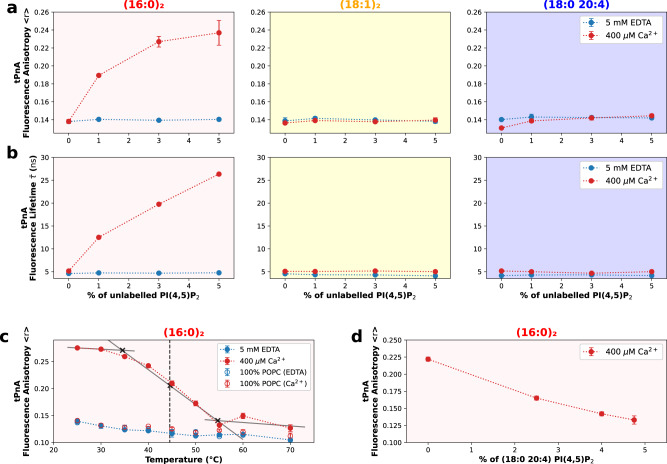
(16:0)_2_ PI(4,5)P_2_ forms gel-like nanodomains upon undergoing calcium-induced clustering. PI(4,5)P_2_ gel-like properties were detected through the incorporation of tPnA at a 1:300 lipid ratio in MLVs containing POPC and increasing concentrations of unlabeled PI(4,5)P_2_. Experiments were done for the three acyl-chain compositions in study. tPnA fluorescence anisotropy (**a**) and fluorescence intensity weighed lifetime ($\overline \tau$) (**b**) were measured in the presence (400 µM Ca^2+^, red) and absence (5 mM EDTA, blue) of calcium. In the presence of calcium, we observed no effect on tPnA fluorescence lifetime for both (18:1)2 PI(4,5)P_2_ (F(1,12) = 1.564, *p* = 0.2349) and (18:0 20:4) PI(4,5)P_2_ (F(1,12) = 0.7426, *p* = 0.4057). For (16:0)2 PI(4,5)P_2_ in the presence of calcium, we observe a steep increase in both fluorescence anisotropy and fluorescence lifetime (F(1,12) = 708.1, *p* ≤ 0.0001). The thermal profile of tPnA anisotropy (**c**) was measured in samples composed of POPC:PI(4,5)P_2_ 90:10. Controls were also carried out with samples of pure POPC. The impact of increasing concentrations of (18:0 20:4) PI(4,5)P_2_ on the formation of (16:0)_2_ PI(4,5)P_2_ gel-like nanodomains (**d**) was detected in MLVs, containing POPC: PI(4,5)P_2_ (95:5) at several (16:0)_2_ to (18:0 20:4) PI(4,5)P_2_ ratios, through the incorporation of tPnA at a 1:300 lipid ratio. Error bars for fluorescence anisotropy measurements represent the SD from *N* = 3 independent experiments.

As saturated phospholipids with (16:0)_2_ acyl chains, such as DPPC, are prone to the formation of solid-like gel phases at room temperature, we aimed to clarify whether that was the case for Ca^2+^-induced (16:0)_2_ PI(4,5)P_2_ nanodomains, as this would explain both the dramatic increase in membrane order and the increase in membrane thickness detected by AFM. To that end, we performed experiments with the membrane probe *trans*-parinaric acid (tPnA). tPnA is the ideal membrane probe for detection of gel lipid domains, as it is one of a few known to exhibit preferential partition to these phases^32^. Large increases in both its anisotropy and fluorescence lifetime are typical hallmarks of gel-phase-like behavior. These parameters were measured in MLVs composed of POPC and increasing concentrations of the different species of PI(4,5)P_2_ (Fig. 4a).

In the absence of calcium, tPnA anisotropy did not respond to increasing concentrations of PI(4,5)P_2_, for any of the studied acyl-chain compositions. This was expected, as the previous experiments indicated that in the absence of calcium, PI(4,5)P_2_ is homogenous and monodisperse. In the presence of calcium, we observed no effect for both (18:1)_2_ PI(4,5)P_2_ and (18:0 20:4) PI(4,5)P_2_. Again, this was expected, as the previous experiments did not hint at these nanodomains having gel-like properties. However, for (16:0)_2_ PI(4,5)P_2_ in the presence of calcium, we observe a steep increase in both fluorescence anisotropy and fluorescence lifetime (Fig. 4a, b), confirming that Ca^2+^-induced (16:0)_2_ PI(4,5)P_2_ nanodomains have a gel-phase-like behavior.

The formation of (16:0)_2_ PI(4,5)P_2_ gel-like phase in the presence of physiologically relevant concentrations of calcium is reminiscent of the previously observed formation of gel-like phosphatidylserine domains by Ca^2+^-induced crosslinking of the anionic phosphoserine (PS) headgroups^33^. In the case of PS, gel formation only occurs at considerably higher calcium concentrations, which limits the biological relevance of this phenomena for that phospholipid. On the other hand, the higher affinity of PI(4,5)P_2_ for the divalent cation promotes the occurrence of the phenomena within the range seen here. Nevertheless, the formation of a gel-like phase of PI(4,5)P_2_ at low lipid concentrations is particularly surprising given the much larger headgroup, which poses greater steric hindrance in the organization of the gel crystal. In addition, the charge density is much higher for PI(4,5)P_2_, leading to greater repulsion between the headgroups. The thermotropic behavior of (16:0)_2_ PI(4,5)P_2_ in the presence of calcium was also tested (Fig. 4c). Unlike the observation made with TMA-DPH, in this case, an inflexion point is clearly visible on the anisotropy data, reflecting a transition from gel-like to fluid phase. Interestingly, the transition temperature of (16:0)_2_ PI(4,5)P_2_ to the fluid phase (*T*_m_) (obtained from the midpoint of the *<r*>_tPnA_ transition in Fig. 4c) occurs at 45 °C, a temperature significantly higher than the one of the corresponding phosphatidylcholine, DPPC (41 °C).

The differences between the data obtained from tPnA and TMA-DPH reflect the absence of partition of TMA-DPH into gel phases. Hence, although tPnA data reflects gel domains specifically, TMA-DPH fails to incorporate into these and will only probe PI(4,5)P_2_ nanodomain environments that failed to transition to the gel phase, possibly due to high local POPC content.

Interestingly, the presence of (18:0 20:4) PI(4,5)P_2_ has a clear impact in the formation of (16:0)_2_ PI(4,5)P_2_ gel-like domains, as seen from Fig. 4d. When equal amounts of both PI(4,5)P_2_ species were present, the fluorescence anisotropy value of tPnA dropped significantly, reflecting a decrease in the extent of gel-like phase formation. In addition, when the canonical polyunsaturated PI(4,5)P_2_ is present at a higher concentration than (16:0)_2_ PI(4,5)P_2_, the formation of PI(4,5)P_2_ gel-like nanodomains is completely abrogated. This result showcases the importance of (18:0 20:4) PI(4,5)P_2_ as an essential modulator of PIP2 nanodomain order, by decreasing the order within PI(4,5)P_2_ nanodomains and thus maintaining their fluidity.

In summary, the results strongly point to two levels of ordering of the Ca^2+^-induced PI(4,5)P_2_ nanodomains above that of the bulk membrane. The formation of a gel-like phase reported by tPnA is unique to saturated PI(4,5)P_2_ species and the corresponding *T*_m_ is higher than that of DPPC, suggesting that the PI(4,5)P_2_ gel-like phase is even more stable than that of PC. On the other hand, a general local ordering reported by TMA-DPH is detected for all PI(4,5)P_2_ species, including those polyunsaturated, and this ordering is not disrupted by temperature in the range evaluated here. The extent of this ordering is directly related to the level of acyl-chain unsaturation of PI(4,5)P_2_.

These results are surprising given the large lipid headgroup in play. Biophysical properties such as membrane hydration, thickness, and flexibility within PI(4,5)P_2_ nanodomains are expected to differ considerably from the rest of the membrane. Such changes are likely to have an impact in membrane events associated to PI(4,5)P_2_. More ordered domains are expected to influence PI(4,5)P_2_ headgroup accessibility, affecting lipid–protein interactions at the core of PI(4,5)P_2_ role in the inner leaflet. In addition, PI(4,5)P_2_ clusters have already been shown by us to prefer partitioning to more ordered domains^14^. It is likely that differences in acyl-chain saturation also regulate the distribution of PI(4,5)P_2_ nanodomains within the plasma membrane.

### CG MD simulations showcase the effects of the different PI(4,5)P_2_ nanodomains on lipid bilayer structure and dynamics

**Fig. 5:**
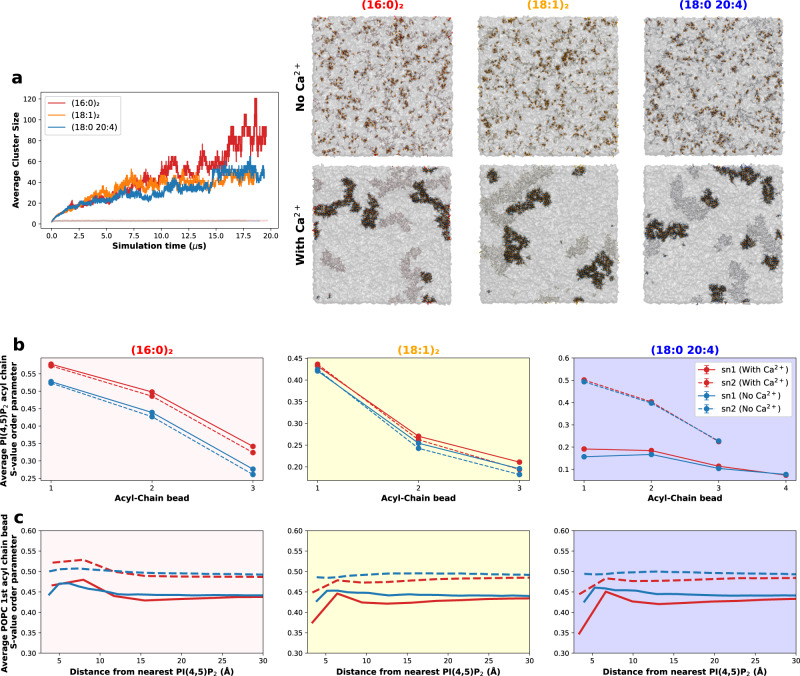
CG MD simulations showcase the impact of acyl-chain composition on PI(4,5)P_2_ and PI(4,5)P_2_ nanodomain biophysical properties. **a** Average PI(4,5)P_2_ cluster size over the course of the simulation for the three acyl-chain compositions studied, both in the presence and absence of calcium. Final simulation snapshots of the large membrane systems are also shown. PI(4,5)P_2_ lipid headgroups are depicted in gray, with the phosphates discriminated in orange. PI(4,5)P_2_ acyl chains are colored according to the corresponding color code. Ca^2+^ ions are represented in blue. The bulk POPC lipids are represented by the translucent gray surface. **b** PI(4,5)P_2_ acyl-chain S-value order parameter for all acyl-chain beads of each composition, in the presence (red) and absence (blue) of calcium. **c** Dependency of the S-value order of POPC’s first acyl-chain bonds on the distance from the nearest PI(4,5)P_2_ molecule, in the presence and absence of calcium.

To gather more structural information on the influence of the acyl-chain composition on the structure and dynamics of these nanodomains, we performed CG MD simulations using the Martini forcefield^34^. These CG MD simulations allow us to study not only PI(4,5)P_2_ and PI(4,5)P_2_ nanodomain dynamics, but also the effects of these nanodomains on the overall biophysical membrane properties. To this effect, large 50 × 50 nm^2^ membrane patches containing 10% PI(4,5)P_2_ were simulated, in the presence and absence of calcium, for at least 19 μs, for the three acyl-chain compositions. These membrane systems were built with 10% PI(4,5)P_2_ to mimic the high concentrations of this lipid in the local environment around cation-induced PI(4,5)P_2_ nanodomains. The last 2 μs of each simulation were considered for analysis (Fig. 5).

Analyzing the dynamics of Ca^2+^-induced nanodomain formation for each composition, through the average PI(4,5)P_2_ cluster size over the course of the simulation (Fig. 5a), we observed little difference between acyl-chain compositions. Nanodomain formation, at least at saturating divalent cation concentrations, seems to depend only on the probability of PI(4,5)P_2_ molecules finding one another, while chelating a calcium ion. This is due to the slow PI(4,5)P_2_-Ca^2+^ and PI(4,5)P_2_-Ca^2+^-PI(4,5)P_2_ unbinding dynamics, caused by the extremely high affinity of the divalent cation for the negatively charged lipid headgroup^35^. Thus, the rate-limiting step of PI(4,5)P_2_ aggregation, in saturating divalent cation concentrations, is lipid diffusion. As all compositions show similar diffusion coefficients (Supplementary Fig. 5D), they end up yielding similarly sized clusters with similar formation dynamics.

Looking at how nanodomain formation influences the biophysical properties of each PI(4,5)P_2_ species, we observed an increase in lipid acyl-chain order (seen through the increase of the S-value parameter, which is described in detail in Supplementary Information) for all three compositions (Fig. 5b). This increase in order, however, is lower for the polyunsaturated and monounsaturated compositions, whereas the results for (16:0)_2_ PI(4,5)P_2_ show a considerable increase in the order parameter (~10%, at the first acyl-chain beads). For the (16:0)_2_ and (18:1)_2_ species, the increase in membrane order is transmitted up to the last acyl-chain bead, where an increase in order is still observed. For the polyunsaturated composition, however, nanodomain formation results in only minor increases in acyl-chain order. In fact, the stearoyl acyl-chain of this PI(4,5)P_2_ species is barely affected by the formation of these nanodomains (1% increase) and the arachidoyl acyl chain only senses this increase in membrane order in the first two beads before dissipating. These results appear to suggest that the 18:0 20:4 composition can better disperse the increase in membrane order, caused by the organization of the PI(4,5)P_2_ headgroups during nanodomain formation and, thus, can better maintain membrane fluidity. Overall, these findings are in excellent agreement with the previous fluorescence data that showed an increase in PI(4,5)P_2_ local membrane order across all the acyl-chain compositions studied, with a particularly high increase for the fully saturated composition, and more moderate changes for the polyunsaturated species.

In addition to the acyl-chain order, we also looked at the extent of their organization. As lipids become tightly packed, they become more structurally organized by extending and straightening their acyl chains in a geometric hexagonal disposition, creating a tightly packed unit with increased van der Waals interactions. These highly ordered hexagonal structures are hallmarks of the formation of gel phases^36,37^. To this purpose, we measured the average number of PI(4,5)P_2_ acyl chains within hexagonal acyl-chain lattices (Supplementary Fig. 5B). As expected, in the absence of calcium, none of the PI(4,5)P_2_ conformations form hexagonal acyl-chain lattices. However, in the presence of calcium, we observe an increase in acyl-chain hexagonality for the monounsaturated composition and a particularly drastic increase for the fully saturated composition. This effect is minimal for the polyunsaturated acyl-chain composition. These findings suggest that cation-induced PI(4,5)P_2_ nanodomains formed from more saturated acyl-chain compositions exhibit not only higher order but also higher organization and packing. For the (16:0)_2_ composition, one could expect that these structures could eventually act as nucleation points for the formation of gel or ripple phases.

The effects of these nanodomains are not merely confined to the PI(4,5)P_2_ lipids within. We found that the nanodomains also affected the order of the surrounding lipids, especially those in the immediate vicinity of the nanodomains. For the poly- and monounsaturated compositions, the domains formed led to a decrease in acyl-chain order of the POPC lipids within 10 Å of PI(4,5)P_2_ (Fig. 5c). This is likely the result of POPC having to accommodate the increased density of unsaturated acyl chains at the border of the PI(4,5)P_2_ nanodomains. Interestingly, we observed the opposite effect for the fully saturated composition. The nanodomains formed with the fully saturated PI(4,5)P_2_ led to an increase in acyl-chain order for POPC lipids within 10 Å of PI(4,5)P_2_. For all compositions, this effect dissipates for POPC lipids further away from the nanodomains.

**Fig. 6:**
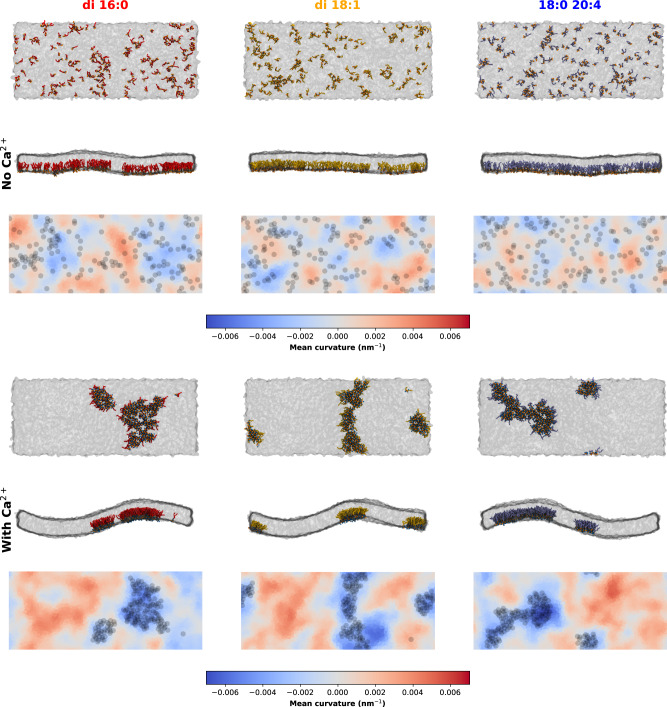
Calcium-induced PI(4,5)P_2_ clustering induces negative membrane curvature, as seen by CG MD simulations. Curvature analysis of snapshots from asymmetric membrane simulations containing 10 mol% PI(4,5)P_2_ in the inner leaflet. Snapshots are shown for the three acyl-chain compositions studied, both in the presence and absence of calcium. For each system, a top and side view are presented, as well as a top view colored by local mean curvature value. PI(4,5)P_2_ phosphodiester (PO4) beads are represented by the black circles. In the molecular representations, PI(4,5)P_2_ headgroups are depicted in gray, with the phosphates discriminated in orange. PI(4,5)P_2_ acyl chains are colored according to the corresponding color code. Ca^2+^ ions are represented in blue. The bulk POPC lipids are represented by the translucent gray surface.

Curiously, we have found that the PI(4,5)P_2_ nanodomains were strongly unregistered (decoupled) across the bilayer (Fig. 5a and Supplementary Fig. 5A). This may be caused by local membrane curvature/tension in the vicinity of Ca^2+^-induced nanodomains, even though curvature was being restricted by an applied potential. As such, we prepared new systems designed to study membrane curvature during PI(4,5)P_2_ nanodomain formation. To this effect, 50 × 20 nm^2^ asymmetric membrane patches, containing 10 mol% PI(4,5)P_2_ in the bottom leaflet only, were simulated for up to 10 μs, for the three acyl-chain compositions (Fig. 6). In the absence of calcium, no PI(4,5)P_2_ clustering occurs and no noticeable membrane undulations are observed.

When calcium was added to the simulations, consolidating PI(4,5)P_2_ nanodomains quickly became associated with a markedly negative curvature for all PI(4,5)P_2_ species (Fig. 6). There were no immediate apparent differences in the curvature generated by the three PI(4,5)P_2_ species. These results are of particular importance, given the requirement of PI(4,5)P_2_ for membrane-remodeling events requiring the formation of highly negatively curved intermediates^3,4,24,25^. As PI(4,5)P_2_ itself is associated with positive curvature, as a result of the bulky headgroup, the presence of PI(4,5)P_2_ within nanodomains could be responsible for alleviating tension on local curvature. Other Martini CG studies have found that inducing membrane curvature, by applying sufficient lateral pressure, could promote the enrichment of PI(4,5)P_2_ at negatively curved membrane areas in the absence of Ca^2+^^38^. However, in our systems without induced curvature, PI(4,5)P_2_ alone was not sufficient to generate curvature undulations, and required Ca^2+^-induced aggregation to associate with negative curvature.

**Fig. 7:**
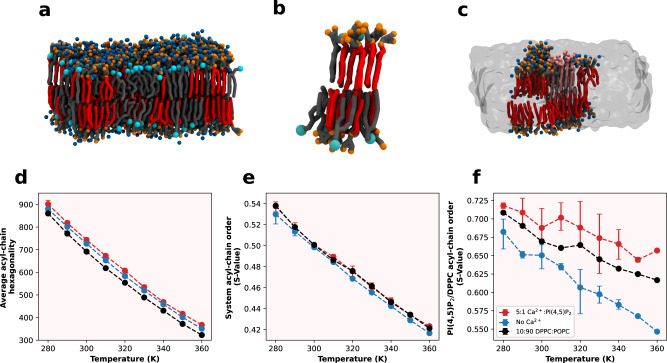
CG MD study of (16:0)_2_ PI(4,5)P_2_ gel-phase nanodomains. **a** Final snapshot of a gel-forming simulation containing 50% mol PI(4,5)P_2_ at 280 K. PI(4,5)P_2_ gel crystals (**b**) were obtained from this simulation and used for the crystal scaffold simulations (**c**), which were used to characterize gel-phase properties. **d** Average system-wide acyl-chain hexagonality dependency with temperature for each of the gel-phase crystal scaffold systems studied. **e** Average system-wide S-value order parameter, calculated on the first acyl-chain bead of every lipid acyl chain, for each of the gel-phase crystal scaffold systems studied. **f** S-value order parameter for the first acyl-chain bead of every gel-forming lipid acyl chain for each of the systems studied. Error bars for all measurements represent the SD from *N* = 3 independent simulation experiments.

To get further structural insight on the formation of gel-like nanodomains of (16:0)_2_ PI(4,5)P_2_ in the presence of calcium, we performed additional simulations in an attempt to detect the formation of gel-like domains in smaller 500 lipid systems. Gel phase-like nanodomains were obtained, albeit at higher PI(4,5)P_2_ mol% (50 mol%) and at lower temperatures (280 K or 6.85 °C) (Fig. 7a) than the experimental conditions. These domains were PI(4,5)P_2_-rich, but contained some POPC molecules incorporated in their structure. The underestimation of the gel-fluid lipid phase transition temperature is well known for Martini forcefield, as seen for the canonical case of DPPC^39^. In addition, it is known that small amounts of disorder-inducing membrane components (such as POPC) are sufficient to disrupt gel-like phases in Martini systems^40^.

To overcome the kinetic barrier posed by the formation of a stable nucleation patch, which slows down the study of the formation of gel phases in Martini, we applied a restrained crystal seeding technique. Briefly, we placed a PI(4,5)P_2_ gel crystal, obtained from one of the previous gel-forming simulations (Fig. 7b), and restrained it in the middle of a membrane system (Fig. 7c). This restrained crystal acts as a stable nucleation point, which we can use to probe the gel growth phase for lower PI(4,5)P_2_ mol% and higher temperatures (Fig. 7c). Using this strategy, the nucleation is shown to induce a consolidation of (16:0)_2_ PI(4,5)P_2_ gel phase formation for the lipid concentration range employed in the experimental studies (10 mol%) (Fig. 7c). In these systems, we followed the average first acyl-chain bead S-value, which gave us an idea of the order of the gel, as well as the hexagonality of the system, which hints at the number of lipids in the gel lattice. The results were compared against a DPPC system built in the same manner.

Looking at the S-value order parameter over a temperature gradient, calculated exclusively from the gel-forming lipid (PI(4,5)P_2_ or DPPC) (Fig. 7f), no clear inflexion point is observed for any of the systems. This is due to the restrained gel crystal in all systems, which prevents the gel phase from completely disappearing, by allowing for the transient incorporation of unrestrained lipids in the crystal. Regardless, PI(4,5)P_2_ in the presence of calcium presents a higher S-value than that of DPPC in the same conditions. This confirms the experimental evidence from tPnA fluorescence, indicating that that the gel domains formed by PI(4,5)P_2_ are more ordered than the canonical gel-forming lipid DPPC. From the slope of this curve, we can infer the thermal stability of these gel domains and, once again, we observe that the domains formed by PI(4,5)P_2_ in the presence of calcium are more stable than those formed by DPPC in the same conditions.

Analyzing the system-wide hexagonality (Fig. 7d), we observe that both PI(4,5)P_2_ systems lead to higher hexagonality values, regardless of the presence of calcium, when compared to DPPC. This result hints that PI(4,5)P_2_ must have a higher propensity to organize itself and the surrounding lipids into a hexagonal lattice. This suggests that the interactions between PI(4,5)P_2_ headgroups, further stabilized by Ca^2+^, lead to a headgroup organization that enhances gel formation.

Within the plasma membrane, an increase of (16:0)_2_ PI(4,5)P_2_ levels in a context of elevated calcium is likely to give rise to gel-like domains in the inner leaflet, which would not only influence PI(4,5)P_2_ lateral organization and availability for interaction with other molecular partners but also potentially impact local membrane properties, such as curvature, thickness, and permeability. Concentrations of Mg^2+^ within mM levels have been shown by us to induce PI(4,5)P_2_ clustering as well^14^, and although not explored in this work, it is likely that high free Mg^2+^ levels, such as those observed intracellularly, might generate similar saturated PI(4,5)P_2_ gel-like domains. If that is the case, then the formation of saturated PI(4,5)P_2_ gel-like nanodomains will be constitutive and independent of calcium levels, with unforeseen consequences.

## Discussion

By investigating representative acyl-chain compositions of the spectrum observed in vivo, we report the effect of acyl-chain saturation on the calcium-dependent changes on the overall properties of PI(4,5)P_2_-containing membranes. In the absence of divalent cations, all acyl-chain compositions behave in the same manner. However, for cation-induced PI(4,5)P_2_ nanodomains, it is clear that the acyl-chain compositions yield structures with significantly different biophysical properties. The key observation is that increasing saturation yields more ordered and structured nanodomains, which can, in the case of the fully saturated composition, culminate in the formation of gel-like nanodomains. Although calcium-dependent gel-like (16:0)_2_ PI(4,5)P_2_ nanodomains are shown to have a melting temperature of ~45 °C, the increase in general local ordering reported for all PI(4,5)P_2_ acyl chains (in the presence of calcium) could not be disrupted by the temperatures tested here (up to 70 °C). The extent of this ordering was directly related to the level of acyl-chain unsaturation of PI(4,5)P_2_.

The increased membrane order detected within calcium-dependent PI(4,5)P_2_ nanodomains is likely to have a profound impact on PI(4,5)P_2_ signaling and on the function and organization of effector proteins. Remarkably, the formation of this phase was largely abrogated in the presence of 1-stearoyl-2-arachidonyl PI(4,5)P_2_. Our findings thus provide an explanation of why evolution strongly favored the 18:0 20:4 acyl-chain composition for PI(4,5)P_2_ in mammalian cells. The presence of the polyunsaturated acyl-chain is expected to guarantee not only low bending rigidity, but also that the ordering within PI(4,5)P_2_ nanodomains remains low. On the other hand, the saturated acyl-chain in the *sn*-1 position could be important to reduce membrane permeability^20^.

Another possible outcome of the formation of ordered calcium-induced PI(4,5)P_2_ clusters is that interactions with proteins exhibiting lower binding affinity for the phospholipid could be switched-off, whereas proteins forming a tighter complex with PI(4,5)P_2_ remain bound. In fact, both the presence of calcium as the modified membrane structure are expected to have an impact on PI(4,5)P_2_–protein interactions. In this scenario, the triggering of PI(4,5)P_2_ clustering could offer an additional level of regulation for PI(4,5)P_2_ signaling.

The molecular simulations carried out here were in clear agreement with the experimental data. Moreover, the simulations identified that the more unsaturated PI(4,5)P_2_ species were more effective in dissipating the increase in order upon clustering over the length of their acyl chains. In addition, simulations also showed that the increase in membrane order was not limited to PI(4,5)P_2_ domains, but extended to the surrounding lipids as well.

We also report an association of calcium-dependent PI(4,5)P_2_ nanodomains with negative curvature, a phenomenon that is likely to play a role in membrane-remodeling events, directly regulated by the presence of PI(4,5)P_2_. These results suggest that the monodisperse PI(4,5)P_2_–PI(4,5)P_2_ nanodomain relation might play a key role in controlling the formation of fusion intermediates required for some of the membrane-remodeling events associated with this phospholipid.

## Methods

### Materials

POPC, DOPC, 1,2-dioleoyl-*sn*-glycero-3-phospho-(1’-*myo*-inositol-4’,5’-bisphosphate) (di18:1 PI(4,5)P_2_), 1-stearoyl-2-arachidonoyl-*sn*-glycero-3-phospho-(1’-*myo*-inositol-4’,5’-bisphosphate) (18:0 20:4 PI(4,5)P_2_), 1-oleoyl-2-{6-[4-(dipyrrometheneboron difluoride)butanoyl]amino}hexanoyl-*sn*-glycero-3-phosphoinositol-4,5-bisphosphate (TopFluor PI(4,5)P_2_), and 1,2-dioleoyl-*sn*-glycero-3-phosphoethanolamine-N-(cap biotinyl) (biotinylated DOPE) were purchased from Avanti Polar Lipids (Alabaster, AL, USA). 1,2-Dipalmitoyl-*sn*-glycero-3-phospho-(1’-*myo*-inositol-4’,5’-bisphosphate) (di16:0 PI(4,5)P2) was from Echelon Biosciences (Salt Lake City, UT, USA). Lipid stock solutions were prepared in chloroform, with the exception of the phosphoinositides, which were prepared in chloroform:methanol (MeOH) 2:1 (v/v). Both solvents were obtained from Merck (Darmstadt, Germany) and were of spectroscopic grade. 4-(2-Hydroxyethyl)-1-piperazineethanesulfonic acid (HEPES), ethanol (EtOH), NaCl, Sucrose, EDTA, glucose, and CaCl_2_ were from Sigma-Aldrich (St. Louis, MO, USA). TMA-DPH, tPnA, Rhodamine 110, and Fluo-5N were from Molecular Probes, Invitrogen (Eugene, OR, USA).

### Liposome preparation

MLVs were prepared by solubilization of lipid films. Briefly, the lipid mixtures were prepared from phospholipid stock solutions, dried under a nitrogen flux, and left in vacuum for 3 h to remove traces of solvent. MLVs were then obtained through the solubilization of the lipid films in the appropriate experimental buffer. Freeze–thaw cycles were performed, using liquid nitrogen and a water bath typically set to 60 °C. The thawing temperature used was always above the melting temperature of the lipid with the highest melting temperature in the mixture, to re-equilibrate and homogenize the samples. LUVs were prepared by extrusion of MLVs^41^, at room temperature, using an Avanti Mini-Extruder (Alabaster, AL, USA) and 100 nm pore-size polycarbonate membranes (Whatman, Little Chalfont, UK). Typically, at least 21 passages through the extruder were performed.

Giant unilamellar vesicles (GUVs) were obtained by gel-assisted formation, based on a method previously described^42^. The lipid mixtures were prepared, from stock solutions, in chloroform to a final concentration of 1.5 mM. DOPE-Cap-biotin was included in the mixture^43^ at a biotinylated lipid/total lipid ratio of 1:750,000. A solution of 5% (w/w) polyvinyl alcohol (PVA) (*M*_W_ ~ 145,000 Da) and 280 mM of sucrose was spread in a μ-slide chamber from Ibidi (Munich, Germany) and left to dry for 15 min at 50 °C. The desired lipid mixture was then spread on the PVA surface. The solvent was evaporated for 15 min under vacuum. Afterwards, the appropriate buffer solution was added, allowing for GUV formation for 60 min at room temperature. Then, GUVs were transferred to a μ-slide chamber with the appropriate coating and left to rest for 10 min, to allow for GUV deposition and immobilization. GUVs were immobilized in the μ-slide chamber through binding of the biotinylated lipid to surface avidin, which was previously used to coat the chamber slide. Coating was carried out using a mixture of bovine serum albumin (BSA), BSA-biotin (9:1 mol/mol), and avidin. Ibidi μ-slide chambers were coated with 300 μL of a 0.9 mg ml^−1^ BSA and 0.1 mg mL^−1^ BSA-biotin mixture for 1 h. Afterwards, the chambers were washed with Milli-Q water and covered with a second layer of 300 μL of 0.01 mg mL^−1^ avidin for 1 h. BSA, BSA-biotin, and avidin solutions were prepared with Milli-Q water. Before adding the GUV suspension, the chambers were also washed with Milli-Q water.

Unless indicated otherwise, all liposome samples were suspended in a buffer solution containing 10 mM HEPES, 140 mM NaCl, and either 400 μM CaCl_2_ or 5 mM EDTA. Buffer Ca^2+^ concentrations in the micromolar range were determined using the fluorescent calcium indicator Fluo-5N pentapotassium salt, following the instructions of the manufacturer.

For samples containing TF-PI(4,5)P_2_, the fluorescent lipid probe was added to the lipid film mixtures at a 0.1 mol% ratio. For TMA-DPH and tPnA samples, the fluorescent lipid probe was added to the lipid film mixtures at a 1:300 lipid ratio (0.33 mol% ratio). MLVs were used for fluorescence studies using TMA-DPH and tPnA, where the probe’s anisotropy and fluorescence lifetime were followed. These properties are not influenced by multilamellarity. For studies using TF-PI(4,5)P_2_, however, LUVs were used in order to avoid possible energy transfer events between bilayers, which would complicate the interpretation of homo-FRET results.

### Steady-state fluorescence spectroscopy

Fluorescence measurements were carried out with a SLM-Aminco 8100 Series 2 fluorescence spectrophotometer (Rochester, NY, USA) with double excitation and emission monochromators (MC-400), in right angle geometry. The light source was a 450 W Xe arc lamp and the reference a Rhodamine B quantum counter solution. Quartz cuvettes (0.5 × 0.5 cm^2^) from Hellma (Müllheim, Germany) were used and temperature set to 25 °C. Polarization of excitation and emission light was obtained with Glan-Thompson polarizers. Blank subtraction was taken into account in all measurements.

Steady-state fluorescence anisotropy, *<r*> is defined as^27^:

$$ < r > = \frac{{I_{{{\mathrm{VV}}}} - G \ast I_{{{\mathrm{VH}}}}}}{{I_{{{\mathrm{VV}}}} + 2 \ast G \ast I_{{{\mathrm{VH}}}}}};\,G = \frac{{I_{{{\mathrm{HV}}}}}}{{I_{{{\mathrm{HH}}}}}}$$

where *I*_Vj_ represents the steady-state vertical (parallel, *I*_VV_) and horizontal (perpendicular, *I*_VH_) components of the fluorescence emission with vertically polarized excitation. The *G* factor is measured using the vertical (*I*_HV_) and horizontal (*I*_HH_) components of the fluorescence emission with horizontaly polarized excitation.

### Time-resolved fluorescence spectroscopy

Fluorescence decay measurements were carried out using the time-correlated single-photon timing technique, as described elsewhere^44^. The emission wavelength was selected by a Jobin Yvon HR320 monochromator (Horiba Jobin Yvon, Kyoto, Japan). Then, 0.5 × 0.5 cm^2^ quartz cuvettes from Hellma were used. Blank decays were acquired, and photon counts were negligible. The fluorescence intensity decays were described by a sum of exponentials:

$$i\left( t \right) = \mathop {\sum}\nolimits_i {\alpha _i{{{{{\mathrm{exp}}}}}}\left( { - \frac{t}{{\tau _i}}} \right)}$$

where *α*_*i*_ is the normalized amplitude and $\tau _i$ is the *i*th lifetime component. The amplitude-weighted average lifetime is defined as:

$$\overline \tau = \mathop {\sum}\nolimits_i {\alpha _i\tau _i}$$

Data analysis was performed with the TRFA software (Scientific Software Technologies Center, Minsk, Belarus), based on Levenberg–Marquardt nonlinear least-squares fitting. The goodness of the fit was judged from the experimental *χ*^2^-weighted residuals and autocorrelation plot. In every analysis, *χ*^2^ was below 1.3, and both the residuals and the autocorrelation were randomly distributed around zero.

### Statistical analysis

Statistical analysis was performed for steady-state fluorescence anisotropy results using regular two-way analysis of variance tests. The two factors accounted for were PI(4,5)P_2_ concentration and whether the sample was in the presence or absence of calcium. F-statistics, degrees of freedom, and *p*-values are reported in the manuscript as called for. No post hoc comparisons were performed. Statistical analysis was performed using GraphPad Prism, GraphPad Software, La Jolla California USA.

### SLB preparation

SLBs were formed by the vesicle fusion rupture method^45,46^. Lipids were mixed at the appropriate molar proportions in a round-bottom flask, dried under nitrogen stream, and left overnight in vacuum to remove any traces of chloroform. Lipid mixtures were hydrated in citrate buffer, to neutralize PI(4,5)P_2_ negative charge and promote vesicle adsorption in the negatively charged mica surface. The multilamellar lipid suspension was power sonicated using a Vibra-Cell ultrasonicator (Sonics & Materials, Newtown, CT, USA) for three times, in cycles of 3 min, with pulsed sonication and 3 min resting in ice. The clear lipid suspension was centrifuged in a microcentrifuge Z 233 M-2 (HERMLE Labortechnik, Wehingen, Germany), for 5 min at 16,500 × *g* to remove titanium particles, large vesicles, and debris. After this, 500 µL of a 10× diluted lipid suspension was pipetted onto freshly cleaved mica along with a 3 mM final concentration of CaCl_2_, in a custom-built well. The sample was incubated in a humidity chamber at 60 °C, above all lipids main transition temperature, for a maximum of 60 min. This procedure allows small unilamellar vesicles to adsorb and rupture on the surface of the mica, forming a flat continuous bilayer^45,46^. After incubation, the bilayer was washed 10–25 times with 100 µL of 60 °C HEPES buffer, using a pipette. The washing procedure was performed parallel to the bilayer surface. This ensures that unfused vesicles, either in suspension or deposited on the bilayer surface, are removed. In all samples, the hydrated bilayers were let to cool down at room temperature, enabling phase separation to occur.

### Atomic force microscopy

AFM was performed using a JPK Nanowizard IV (JPK Instruments, Berlin, Germany). Bilayers were imaged in quantitative imaging (QI) mode, a recent innovation in which the apparatus modulates the *z*-piezo to perform a fast force curve on each pixel of the image^47,48^. This avoids lateral friction and allows for better control of the tip force during measurements. QI mode allows several mechanical properties to be calculated from the force applied and the tip-sample separation. Throughout the imaging, the maximum applied force was 200 pN, in order not to affect the sample structure^49^. Images were obtained with a resolution of 256 × 256 pixels, at a scan rate of 1 Hz. AFM measurements were performed at room temperature, which varied from 22 °C to 25 °C.

Before measurements, cantilever spring constants were quantified by the thermal noise method^50^ and cantilever sensitivity was measured by performing a force curve on a clean freshly cleaved mica surface, in HEPES buffer. For the used qpBioAC CB2 probes (NanoWorld AG, Neuchâtel, Switzerland), the spring constants obtained were on the 0.06–0.18 N m^−1^ range and the sensitivity 7.6 ± 1.2 nm V^−1^. Approximately three to five separated areas, of 10 × 10 μm^2^ and 20 × 20 μm^2^, from at least two bilayers, prepared in different days, from different lipid stocks, were imaged to obtain representative data and assure the reproducibility of the measurements.

Topographical images were analyzed with first or second-level flattening, using the JPK data processing software. The sizes of the domains were evaluated by several cross-sections on the SLB images. The number of cross-sections varied from 70 to ~300, to obtain representative data for the domains formed in each SLB.

### MD simulations

The Martini 2.2 CG model for biomolecular simulations was employed throughout this study^34^. The used PI(4,5)P_2_ topologies were constructed combining an in-house improved version of the existing PI(4,5)P_2_ headgroup parameters^51^ with the standard Martini lipid acyl-chain topologies for each of the compositions studied. The rationale behind the improved PI(4,5)P_2_ headgroup parameters is described in detail in the Supplementary MMethods (Supplementary Figs. 7–11). These topologies are provided, in the standard GROMACS format, in Supporting Information (PIP2.itp) and are also available from the authors. All other topologies were obtained directly from the Martini developers’ website^52^. All simulations were run using the GROMACS simulation package version 2018^53^. The membrane systems were built and solvated using the *insane.py* CG building tool^54^. Counterions were added to neutralize the system as necessary and 140 mM NaCl was added on top of that to all systems. When required, Ca^2+^ was added at the appropriate Ca^2+^:PI(4,5)P_2_ ratio by replacing water particles and maintaining the system charge neutral by adding Cl^−^ counterions.

Nonbonded interactions were cutoff at 1.1 nm and Coulombic interactions were treated using reaction-field electrostatics^55^. The particle neighbor list was updated using the Verlet list scheme. The v-rescale thermostat^56^ was used with a tau-t of 4.0 ps to maintain the temperature at 300 K. Constant pressure was semi-isotropically coupled to 1.0 bar using a Parrinello–Rahman barostat^57^ with a relaxation time of 16.0 ps. After initial energy minimization and pressure/temperature equilibration runs, simulations were run at a 20 fs time step.

Three types of systems were simulated as follows:

*— large* systems consisting of ~8500 lipids at a 90:10 POPC:PI(4,5)P_2_ molar ratio in a 50 × 50 × 13 nm^3^ box were simulated for at least 19 μs. Simulations were ran for each composition both in the presence and absence of Ca^2+^. For these systems only, membrane undulations were limited by applying a weak (200 kJ mol^−1^ nm^−2^) flat-bottomed potential in *z*, restraining the glycerol beads of all lipids to remain within a 2.0 nm vertical distance of the simulation box center.

*— asymmetric* membrane systems, consisting of ~3500 lipids, in a 50 × 20 × 13 nm^3^ box, which were simulated for at least 10 μs. Their top membrane layer consists solely of POPC, whereas the bottom layer consists of a 90:10 POPC:PI(4,5)P_2_ mixture. The total number of lipids in each lipid layer was adjusted, matching the top and bottom leaflet areas, thereby avoiding the introduction of tension. To this end, the area per lipid of each layer’s composition was calculated from smaller symmetric simulations ran for each of the compositions, both in the presence and absence of Ca^2+^.

— *crystal seed* systems, where gel-phase formation was probed by simulations of a PI(4,5)P_2_ gel crystal seed restrained in the middle of an initially fluid membrane. Gel-phase crystals were obtained through regular simulations of POPC: PI(4,5)P_2_ mixtures (~500 lipids; 15 × 10 × 13 nm^3^ boxes) with PI(4,5)P_2_ molar ratios ranging from 30% to 50 %, simulated for up to 10 μs. These simulations were run with Ca^2+^, at a 5:1 Ca^2+^:PI(4,5)P_2_, using the same general MD parameters, except for the temperature, which was maintained at 280 K. From these initial simulations, PI(4,5)P_2_-rich gel phases were formed, from which small ~20 lipid crystals were excised. These crystals were inserted in membranes of the same size at a 90:10 POPC:PI(4,5)P_2_ molar ratio, simulated for at least 5 μs. These were run either in the absence or in the presence of calcium, at a 5:1 Ca^2+^:PI(4,5)P_2_ ratio, and at temperatures ranging from 280 to 360 K. Throughout all the simulation steps, the acyl chain and glycerol beads of the crystal seeds were position-restrained in all dimensions with a force constant of 10,000 kJ mol^−1^ nm^−2^. The headgroups were left unrestrained to better accommodate new lipids in the growing crystal. Three replicates, with independent gel-phase crystals, were run for each condition.

All simulations were analyzed making use of in-house developed Python3 programs using the MDAnalysis package^58^. We also used the IPython^59^, numpy^60^, SciPy^61^, scikit-learn^62^, and matplotlib^63^ packages for scientific computing in Python. The PyCurv^64^ package was used for curvature estimation. Visualization and renderization of the simulations was performed with the molecular graphics viewer VMD^65^. The last 2 μs of each simulation were used for analysis. See the “Methods” section of the Supporting Information for details on the analysis methods.

## Supplementary information

Supplementary Information

## Data Availability

Extended Methods and Materials providing details on the rationale behind the improvement of the Martini 2.2 PI(4,5)P2 model, details behind the simulation analysis, additional time-resolved fluorescence spectroscopy results, fluorescence correlation spectroscopy results on TF-PI(4,5)P2 incorporation in nanodomains, AFM analysis of nanodomain sizes, and further simulation analysis of PI(4,5)P2 biophysical properties are available within Supplementary Information. The molecule description file containing the CG parameters for the Martini 2.2 PI(4,5)P_2_ models used in this study in text format compatible with GROMACS software, as well as initial and final configurations for the molecular dynamics simulation systems are available from https://github.com/MeloLab/PhosphoinositideParameterization. Other data are available from the corresponding author upon reasonable request.
