# Supplementary material for: Acyl-chain saturation regulates the order of phosphatidylinositol 4,5-bisphosphate nanodomains
Source: Commun Chem. 2021 Nov 29;4:164. doi: 10.1038/s42004-021-00603-1 (PMC9814227; doi:10.1038/s42004-021-00603-1)
Supplement: Supplementary file 1 — Supplementary Information [file 42004_2021_603_MOESM1_ESM.pdf]

Supporting Information

Acyl-chain saturation regulates the order of phosphatidylinositol 4,5-bisphosphate nanodomains

Luís Borges-Araújo †,‡, Marco M. Domingues §, Alexander Fedorov †, Nuno C. Santos §, Manuel N. Melo‡, Fábio Fernandes †,‖ *

† Institute for Bioengineering and Biosciences (IBB) and Associate Laboratory i4HB-Institute for Health and Bioeconomy, Instituto Superior Técnico, Universidade de Lisboa, Lisbon, Portugal

‡ Instituto de Tecnologia Química e Biológica António Xavier, Universidade Nova de Lisboa, Av. da República, 2780-157 Oeiras, Portugal.

§ Instituto de Medicina Molecular, Faculdade de Medicina, Universidade de Lisboa, Av. Prof. Egas Moniz, 1649-028 Lisbon, Portugal;

‖ Department of Bioengineering; Instituto Superior Técnico, Universidade de Lisboa, 1049-001 Lisbon, Portugal;

* Author to whom correspondence should be addressed; email: fernandesf@tecnico.ulisboa.pt

SUPPLEMENTARY METHODS

Improved Martini 2.2 coarse-grain parameterization of PI(4,5)P_2. With the goal of using the Martini 2.2 coarse-grained (CG) molecular dynamics (MD) forcefield for studies of PI(4,5)P_2 in lipid membranes, the parameters of the phosphatidylinositol (PI) headgroup and of PI(4,5)P_2 were improved from existing parameters[1]. A focus was made on more accurately describing the behavior seen in atomistic simulations and in improving the numerical stability of the topology. The existing PI/ PI(4,5)P_2 parameters had a dihedral potential in place that led to instability at typical Martini timesteps[2]; they also describe the conformational dynamics of the lipid headgroup with insufficient accuracy. The topology further showed an unintended propensity for aggregation, even in the absence of divalent cations. This is not observed experimentally, where only divalent cations have the affinity to induce this kind of lateral reorganization. Part of this behavior could be explained by an overly loose headgroup conformation.

PI was reparameterized in an aqueous solution and in a pure PI membrane as described in López et al. 2013[1]. PI(4,5)P_2 was only parameterized in aqueous solution. The same all-atom (AA) simulations that were used in López et al. 2013[1] were used as reference for the improved parameterization as well. The AA trajectories were converted into CG trajectories using the center of mass of the appropriate AA particles[1]. These backmapped trajectories were then used to obtain the several dihedral, angle and distance distributions, that were here considered as reference. Both the single lipid in water and the membrane systems were built and solvated as described in López et al. 2013[1]. All CG simulations were modelled using the Martini 2.2 CG model for biomolecular simulations[3] and run with GROMACS[4] version 2018. For the CG simulations, nonbonded interactions were cutoff at 1.1 nm and Coulombic interactions were treated using reaction-field electrostatics[5]. The particle neighbor list was updated using the Verlet list scheme. The equations of motion were integrated

using a timestep of 20 fs. The Stochastic Dynamics (SD) integrator was used as the thermostat, with an inverse friction constant of 2.0, maintaining the temperature at 310 K. Constant pressure was maintained at 1.0 bar by coupling to a Berendsen barostat with a relaxation time of 3.0 ps. For the simulations used to test the stability of the topology, the V-rescale thermostat was used, with a tau-t of 1.0, to maintain the temperature at 310 K. As was done in López et al. 2013[1], anisotropic scaling was performed for all bilayer systems, whilst for the aqueous systems isotropic scaling was used. The base topology for PI (DPPI) was obtained from the Martini coarse grain forcefield website[6].

To better match the PI conformations observed in the AA simulations and improve the stability of the topology at longer timesteps, the dihedral, angle and bond potentials were tuned. No bead types were changed from the original topology. The strategy employed was to replace the dihedral that was in place to control the headgroup orientation relative to the glycerol backbone (C3-C1-PO4-GL1) by more robust ones (i.e., less likely to operate over particle angles that can become collinear), and to adjust particle angles to improve overall stability. This was done by defining three dihedrals (C1-PO4-GL1-GL2, C2-C3-GL1-GL2 and C2-C3-C1-PO4), adding 3 new angles (GL2-GL1-C3, C2-C3-GL1 and PO4-GL1-GL2) and adjusting the angles that were already defined (C3-C1-PO4, C2-C1-PO4 and C1-PO4-GL1). For angles that were involved in the dihedrals, and that could reach 180°, a restricted bending potential was used, in place of the regular harmonic angle potential, to avoid numerical instabilities. This new approach better represented the distributions obtained from the backmapped AA simulations (figure S6) and yielded a topology stable in membrane simulations for at least 10µs at both 20 and 30 fs timesteps. The recovered values for the average area per lipid and average membrane thickness, at 310 K, were 0.598±0.004 nm^2 and 4.08±0.02 nm, respectively. These are in agreement with the values obtained by AA simulations, in López et al. 2013, of 0.65nm^2 and 3.9 nm, and with those obtained with the previous CG topology of 0.62±0.004 nm^2 and 4.0 nm.

Since PI(4,5)P_2 is a phosphorylated derivative of PI, the PI CG topology was used as a building block for the parameterization, to which the P4 and P5 phosphates were added. To connect these phosphates to the headgroup, we maintained the strategy that was employed by López et al. 2013[1] of using 2 bonds to inositol headgroup beads and 1 dihedral potential, to control the phosphate orientation relative to the ring plane. For each phosphate, the main bond was set at the appropriate AA distance and the secondary bond was increased slightly in order to control the phosphate angle relative to the inositol ring. This artificial increase in distance for the secondary bond is required due to the also increased distance between the inositol headgroup beads, which was put in place to improve area per lipid values. Contrary to what was done in López et al. 2013[1], harmonic bonds were used instead of constraints to obtain better conformation flexibility (these can eventually be set as constraints if necessary). Additionally, as the two phosphates are placed very close together, an exclusion between them was put in place. Only a slight adjustment to one of the dihedral potentials (C2-C3-C1-PO4) that controls the inositol headgroup (derived from the PI CG topology) was necessary. We were able to accurately represent the phosphate dynamics obtained from the backmapped AA simulations (figure S7). This topology was stable in membrane simulations for at least 50 µs using both 20 and 30 fs timesteps. The recovered values for the average area per lipid and average membrane thickness, at 310 K, were 0.715±0.007 nm^2 and 3.64±0.02 nm, respectively.

As mentioned above, with the original PI(4,5)P_2 topology we observed a propensity for unintended aggregation, especially through the interaction with sodium, which is not observed experimentally. With the improved PI(4,5)P_2 topology we can observe a significantly better propensity for aggregation (figure S8, A and B). This is clearly reflected in the PI(4,5)P_2 vs. PI(4,5)P_2 radial distribution function, where we observe a significant decrease in PI(4,5)P_2 neighbors with the improved topology (figure S8, C). It must be noted that the propensity for PI(4,5)P_2 to undergo

calcium induced clustering, was already present in the original topology and was not introduced by our modifications (figure S10).

Molecular dynamics simulation analysis. All simulations were analyzed making use of in-house developed Python3 programs using the MDAnalysis package. We also used the IPython[7], numpy[8], SciPy[9], scikit-learn[10] and matplotlib[11] packages for scientific computing in Python. Visualization and renderization of the simulations was performed with the molecular graphics viewer VMD[12]. The last 2 μs of each simulation were used for analysis.

— The *S-value order parameter* is a geometric parameter which can be used to compare the different lipid tails' overall order/flexibility. Lipid tail S-value order parameters were calculated for each lipid tail bond, of each independent lipid as described previously[13]. The S-value used here is the extension to coarse-grain of the concept of lipid C-H bond order parameter, and is defined as: $S = \frac{1}{2}(3\langle(\cos\theta)^2\rangle - 1)$, where θ is the angle between the vector along a particular lipid tail bead pair and the normal of the bilayer, which we approximate as the system's z-axis. When calculating the S-Value vs. the distance to a reference target, each S-Value obtained was then binned according to their distance to the reference in the *xy* plane. Bins with less than analyzed 200000 elements over the simulation time (2 μs) were removed from analysis.

—The *hexagonality index* was used in comparing the ability of the different lipid tails to organize into a hexagonal matrix. Hexagonality was calculated for the first lipid tail bead of each individual lipid. A lipid tail bead was considered to be within an hexagonal matrix if the following criteria were met: i) the tail bead is within 6 Å of the 6 closest neighbors; and ii) each of the 6 closest neighbors to the target bead also has at least 2 neighbors within that cutoff. To eliminate some thermal fluctuation noise the hexagonality index was calculated from a trajectory with coordinates averaged over a 5-frame running window.

—*Average area per lipid (APL)* was calculated from the average box area divided by the number of lipids in each leaflet.

—*Lipid lateral diffusion* was calculated from the mean square displacement (MSD) of the molecules in the membrane plane over a range of window sizes: 1, 3, 5, 10, 25, 50, 100, 200, 300, 400, 500 ns. Diffusion constants were then estimated from non-linear least squares fitting to the two parameter equation described previously[14,15]: $MSD = ND_\alpha t^\alpha$, where N represents the degrees of freedom coefficient, which in the case of planar phospholipid bilayers is 2, t represents time and D_α is measured in units of length^2/time$^\alpha$. Standard deviation for both parameters was taken from the square root of the diagonal of the covariance matrix obtained from the non-linear squares fit. Lateral diffusion was calculated for each lipid independently considering the position of its PO4 bead.

—*Radial distribution functions (RDF)* are obtained by processing the distance matrix from one reference set of positions to one or more sets of positions. The distance arrays are histogrammed and averaged over the simulation time. The RDF is then obtained by normalizing the histogram by the number of reference positions, volume of the bin and average particle density of the positions. In our cases the RDFs are calculated over the *xy* membrane plane and thus the *z* component of the positions is not taken into consideration. Additionally, normalization employs the area of the bin instead of the volume.

—*Membrane curvature analysis* was performed using the PyCurv module and adapting the methods described elsewhere[16]. In summary, the PO4 beads from one of the bilayer leaflets were selected and

used to generate a triangulated surface. To correctly calculate the curvature near the periodic boundaries, a neighboring periodic copy of the membrane was added in each of the *xy* directions before surface triangulation. This surface was then processed, and a surface graph generated from which the local surface normal, principal directions and curvatures were estimated. Principal directions and curvatures were estimated using the vector voting (VV) method using a neighborhood of 5 nm. This neighborhood radius was calibrated to better show the features of interest. In general, the neighborhood radius should be set to the radius of the smallest feature of interest on the input surface.

Fluorescence correlation spectroscopy. FFS measurements were performed on a Leica TCS SP5 (Leica Microsystems CMS GmbH, Manheim, Germany) inverted confocal microscope (DMI600). Excitation lines provided by an argon laser were focused into the sample through an apochromatic water immersion objective (63×, NA 1.2; Zeiss, Jena Germany). A 111.4 μm diameter pinhole in front of the image plane blocked out of focus signal. Fluorescence emission was detected using avalanche photodiodes (APDs) after passing through a 500–550 nm band-pass filter. Excitation of TF-PI(4,5)P2 was performed with the 488 nm Ar laser line. Fluorescence fluctuations from GUVs with incorporated fluorescent probes were recorded from the top of the vesicle, with the focal volume centered in the focal plane with maximum fluorescence intensity. Five autocorrelation (AC) curves, G(τ), were sequentially obtained for each sample, with an acquisition time of 20 s at a 100 kHz sampling frequency. Assuming a Gaussian measurement volume, AC curves were fitted using a model accounting for 2D translational diffusion, and [17]:

$$G(\tau) = \frac{1}{C\pi\omega_{xy}^2}\left(1+\frac{4D\tau}{\omega_{xy}^2}\right)^{-1} = \frac{1}{N}\left(1+\frac{\tau}{\tau_D}\right)^{-1}$$

The dimensions of the focal volume were determined by calibration with rhodamine 110 in milliQ water using the same optical setup as the samples. A rhodamine 110 diffusion coefficient (D) of 440 $\mu m^2 s^{-1}$ was considered[18]. Analysis of all the FCS experimental data was carried out using the ISS Vista software. This program employs a Levenberg–Marquardt nonlinear least-squares fitting routine and the goodness of the fittings can be judged by the recovered χ^2 value and the random distribution of the weighted residuals.

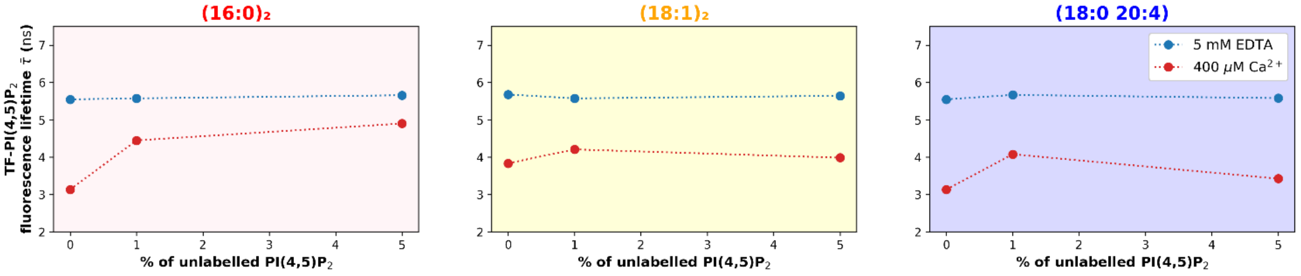

Supplementary Figure 1. Cation-induced clusters are formed independently of PI(4,5)P2 acyl-chain composition as seen by homoFRET of the TF- PI(4,5)P2 analogue. PI(4,5)P2 cluster formation was determined through the incorporation of 0.1% of TF- PI(4,5)P2 in 100 nm LUVs containing POPC and increasing concentrations of unlabeled PI(4,5)P2. The experiments were carried out for the three PI(4,5)P2 species in study and figures are labeled according to the acyl-chain composition of the unlabeled PI(4,5)P2 employed. TF-PI(4,5)P2 fluorescence intensity weighed lifetime ($\bar{\tau}$) was measured in the presence (400 μM Ca^{2+}, red) and absence (5 mM EDTA, blue) of calcium. It should be noted, that since fluorescence anisotropies of fluorophores are inversely dependent on their respective fluorescence lifetimes[17], that the decrease in lifetime observed during calcium dependent clustering of the analogue, leads to a slightly attenuated drop in <*r*> values.

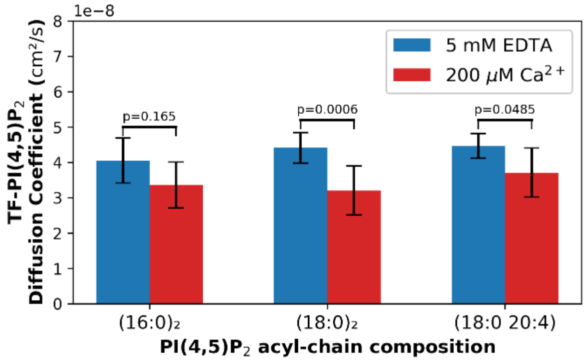

Supplementary Figure 2. TF-PI(4,5)P2 is incorporated into cation-induced PI(4,5)P2 clusters regardless of acyl-chain composition. Diffusion coefficients obtained for 0.01% TF- PI(4,5)P2 in 97.5:2.5 POPC:PI(4,5)P2 GUVs, in the presence and absence of calcium. The experiments were done for the three PI(4,5)P2 species in study and figures are labeled according to the acyl-chain composition of the employed unlabeled PI(4,5)P2. Error bars represent the standard deviation from measurements obtained from at least N=4 independent GUVs.

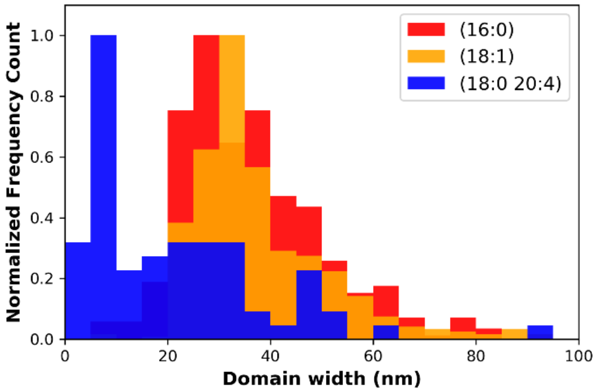

Supplementary Figure 3. PI(4,5)P2 unsaturated acyl-chain compositions appear to promote the formation of smaller nanodomains as seen by AFM. PI(4,5)P2 cluster formation was detected through the AFM measurement of SLBs containing DOPC and 5% PI(4,5)P2 . The experiments were carried out for the three PI(4,5)P2 species in study and the histograms are labeled according to the acyl-chain composition of the PI(4,5)P2 species employed. Topographical images were acquired and analyzed with first or second level flattening, using the JPK data processing software, from which the domain width was determined. The sizes of the domains were evaluated by several cross sections on the topographical images. The number of cross sections varied from 70 to approximately 300 to obtain representative data of the domains formed in each SLB. The frequency count was normalized to the highest bin.

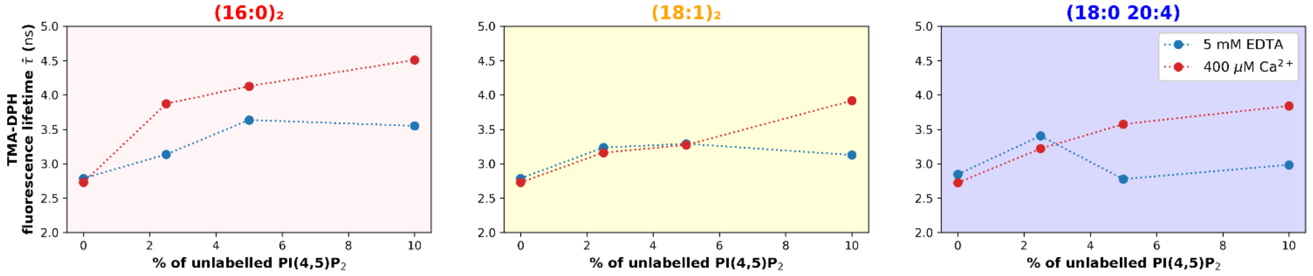

Supplementary Figure 4. TMA-DPH probe incorporation shows that calcium-induced PI(4,5)P2 nanodomains are significantly more ordered than monodisperse PI(4,5)P2 even for unsaturated acyl-chain compositions. PI(4,5)P2 local membrane order was determined through the incorporation of TMA-DPH at a 1:300 lipid ratio in MLVs containing POPC and increasing concentrations of unlabeled PI(4,5)P2. The experiments were done for the three acyl-chain compositions in study. TMA-DPH fluorescence intensity weighed lifetime was measured in the presence (400 μM Ca2+, red) and absence (5 mM EDTA, blue) of calcium.

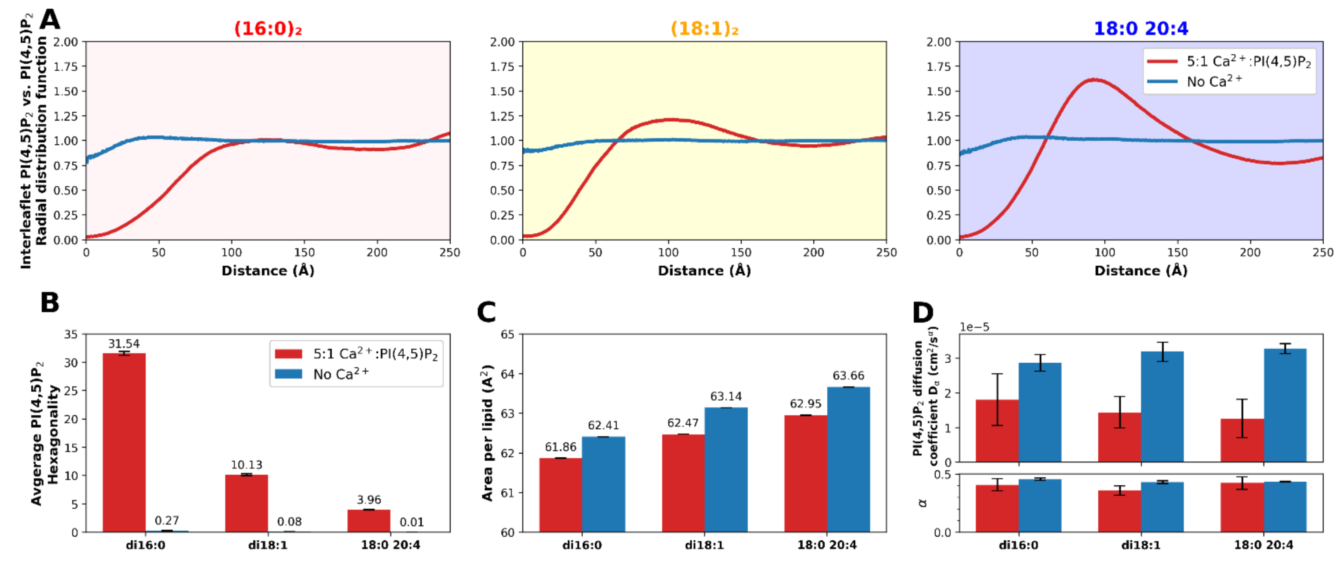

Supplementary Figure 5. CG MD simulations showcase the impact of acyl-chain composition on PI(4,5)P$_2$ and PI(4,5)P$_2$ nanodomain biophysical properties. (A) Interleaflet PI(4,5)P$_2$ vs PI(4,5)P$_2$ radial distribution function for each composition in the presence and absence of calcium. (B) Average PI(4,5)P$_2$ acyl-chain hexagonality for each composition in the presence and absence of calcium. (C) Average membrane area per lipid for each system in the presence and absence of calcium. (D) PI(4,5)P$_2$ diffusion coefficient and scaling exponent recovered from an anomalous diffusion analysis of the simulation, in the presence and absence of calcium.

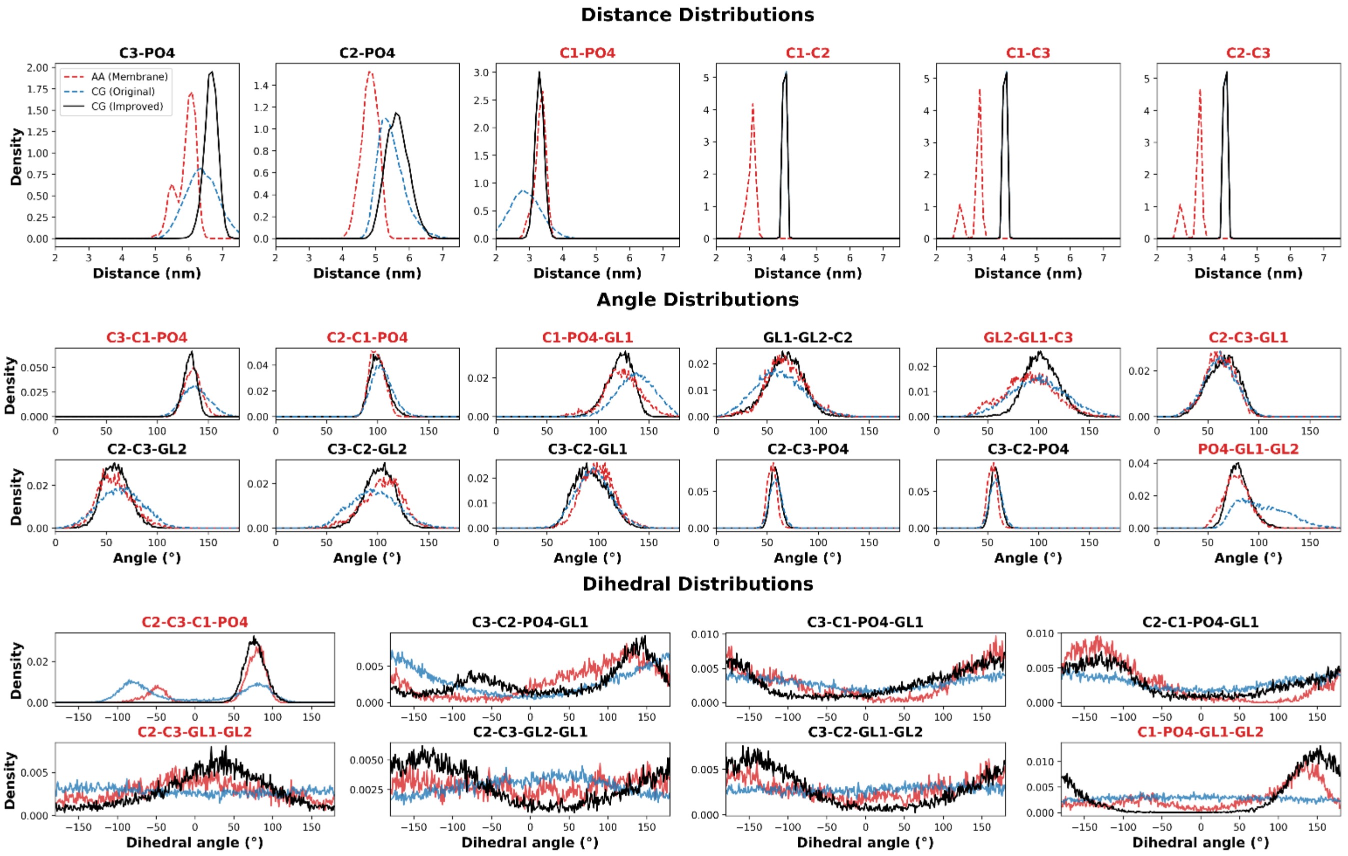

Supplementary Figure 6. Bonded parameters from the original and improved PI topologies. Tracked distance, angle and dihedral distributions from Martini 2.2 PI models as well as the reference atomistic distributions. These were obtained from mapping AA simulations (red line) and compared to the original (blue line) and the improved (black line) CG topologies. The distributions that have an applied potential in the improved topology are marked in red.

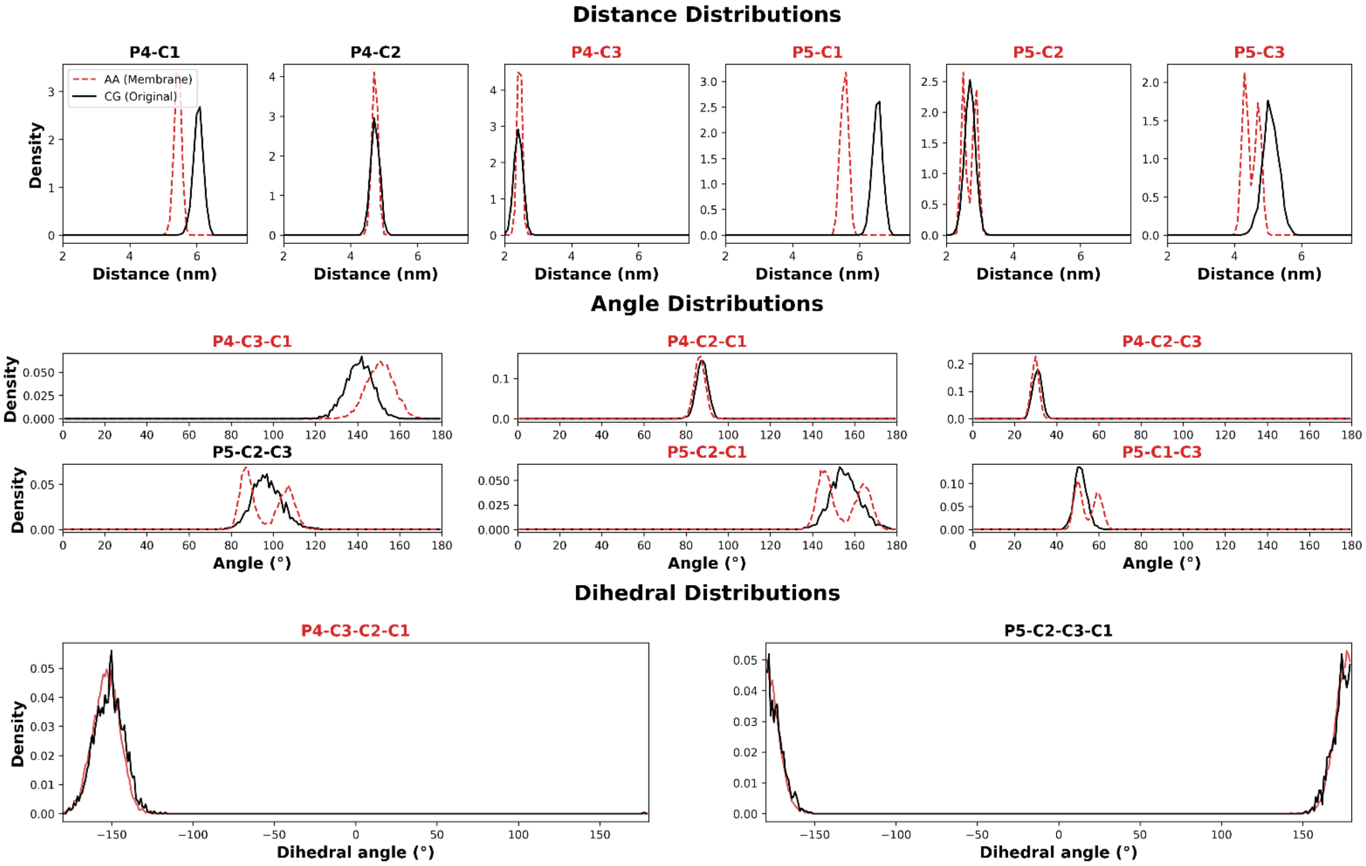

Supplementary Figure 7. Phosphate bonded parameters from the original and improved PI(4,5)P$_2$ topologies. Tracked distance, angle and dihedral distributions of the phosphate particles from Martini 2.2 PI(4,5)P$_2$ models as well as the reference atomistic distributions. These were obtained from mapping AA simulations (red line) and compared to the original (blue line) and the improved (black line) CG topologies. The distributions that have an applied potential in the improved topology are marked in red.

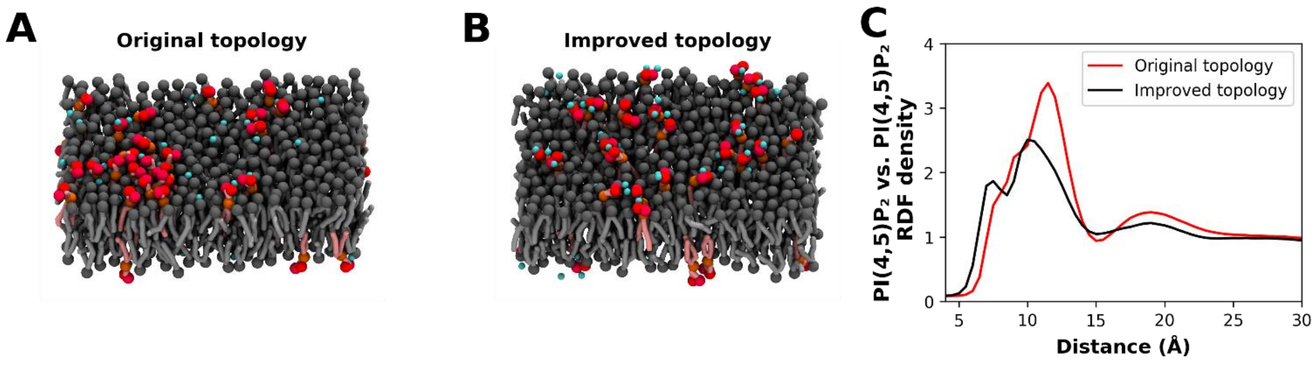

Supplementary Figure 8. Influence of the topology on PI(4,5)P$_2$ non-specific aggregation. Final simulation snapshots of a membrane simulation containing a 90:10 POPC:PI(4,5)P$_2$ lipid mixture in the presence of 140 mM NaCl. This simulation was performed with the original (A) and the updated (B) headgroup parameters and ran for at least 40 µs. POPC is depicted in grey, PI(4,5)P$_2$ in red and sodium atoms within 7 angstroms of PI(4,5)P$_2$ in blue. Radial distribution functions (RDFs) of the PI(4,5)P$_2$ headgroup center of mass against itself (C), calculated from the same membrane simulations. The result obtained by the original and improved parameters are depicted in red and black, respectively.

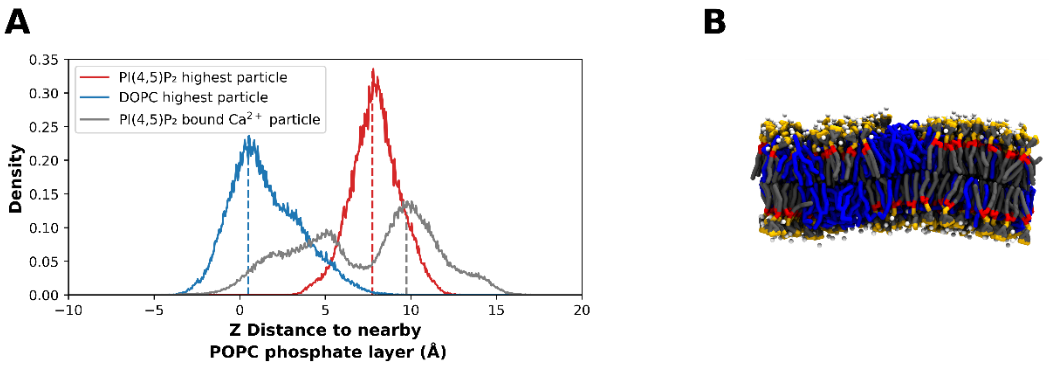

Supplementary Figure 9. Impact of (16:0)$_2$ PI(4,5)P$_2$ on bilayer thickness in fluid CG MD simulations. Density profile (A) and final simulation snapshot (B), of a membrane simulation containing a 50:50 DOPC: (16:0)$_2$ PI(4,5)P$_2$ lipid mixture in the presence of calcium, showcasing the difference in height between (16:0)$_2$ PI(4,5)P$_2$ nanodomains and the bulk membrane.

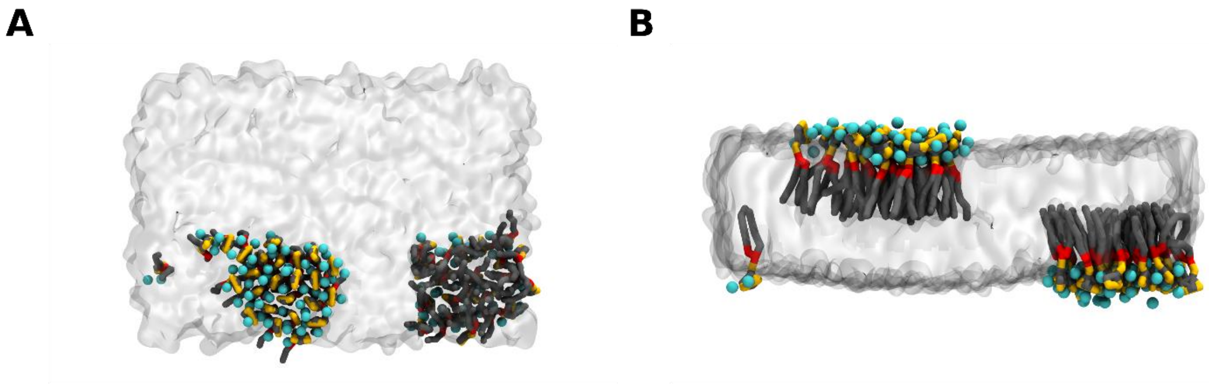

Supplementary Figure 10. Calcium-induced aggregation was already observed for the original PI(4,5)P$_2$ Martini 2 CG MD topology. Final simulation snapshots of a membrane simulation containing a 90:10 POPC: (16:0)$_2$ PI(4,5)P$_2$ lipid mixture in the presence of calcium. This simulation was performed with the original headgroup parameters and ran for 4 µs. PI(4,5)P$_2$ lipid headgroups and acyl-chains are depicted in grey, with the phosphates discriminated in orange and the glycerol backbone in red. Ca^{2+} ions are represented in blue. The bulk POPC lipids are represented by the translucent grey surface.

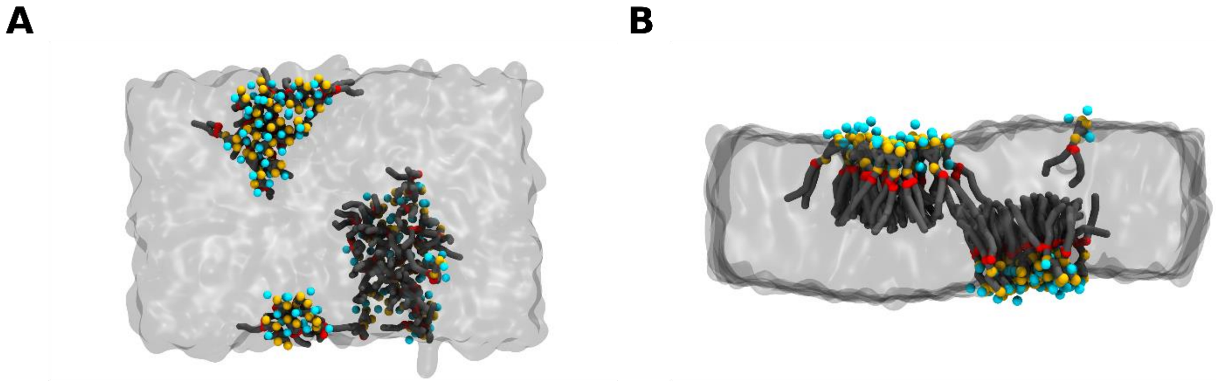

Supplementary Figure 11. Calcium-induced PI(4,5)P$_2$ aggregation is observed with particle mesh ewald (PME) electrostatic treatment. Final simulation snapshots of a membrane simulation containing a 90:10 POPC: (16:0)$_2$ PI(4,5)P$_2$ lipid mixture in the presence of calcium. This simulation was performed with particle mesh ewald electrostatics, the updated PI(4,5)P$_2$ headgroup parameters and ran for 4 µs. PI(4,5)P$_2$ lipid headgroups and acyl-chains are depicted in grey, with the phosphates discriminated in orange and the glycerol backbone in red. Ca^{2+} ions are represented in blue. The bulk POPC lipids are represented by the translucent grey surface.

SUPPLEMENTARY REFERENCES

(1) López, C. A.; Sovova, Z.; van Eerden, F. J.; de Vries, A. H.; Marrink, S. J. Martini Force Field Parameters for Glycolipids. *J. Chem. Theory Comput.* **2013**, *9* (3), 1694–1708.

(2) Bulacu, M.; Goga, N.; Zhao, W.; Rossi, G.; Monticelli, L.; Periole, X.; Tieleman, D. P.; Marrink, S. J. Improved Angle Potentials for Coarse-Grained Molecular Dynamics Simulations. *J. Chem. Theory Comput.* **2013**, *9* (8), 3282–3292.

(3) Marrink, S. J.; Risselada, H. J.; Yefimov, S.; Tieleman, D. P.; De Vries, A. H. The MARTINI Force Field: Coarse Grained Model for Biomolecular Simulations. *J. Phys. Chem. B* **2007**, *111* (27), 7812–7824.

(4) Abraham, M. J.; Murtola, T.; Schulz, R.; Páll, S.; Smith, J. C.; Hess, B.; Lindah, E. Gromacs: High Performance Molecular Simulations through Multi-Level Parallelism from Laptops to Supercomputers. *SoftwareX* **2015**, *1–2*, 19–25.

(5) De Jong, D. H.; Baoukina, S.; Ingólfsson, H. I.; Marrink, S. J. Martini Straight: Boosting Performance Using a Shorter Cutoff and GPUs. *Comput. Phys. Commun.* **2016**, *199*, 1–7.

(6) Martini Coarse Grain Forcefield for Biomolecules http://cgmartini.nl/.

(7) Pérez, F.; Granger, B. E. *IPython: A System for Interactive Scientific Computing*.

(8) Harris, C. R.; Millman, K. J.; van der Walt, S. J.; Gommers, R.; Virtanen, P.; Cournapeau, D.; Wieser, E.; Taylor, J.; Berg, S.; Smith, N. J.; Kern, R.; Picus, M.; Hoyer, S.; van Kerkwijk, M. H.; Brett, M.; Haldane, A.; del Río, J. F.; Wiebe, M.; Peterson, P.; Gérard-Marchant, P.; Sheppard, K.; Reddy, T.; Weckesser, W.; Abbasi, H.; Gohlke, C.; Oliphant, T. E. Array Programming with NumPy. *Nature* **2020**, *585* (7825), 357–362.

(9) Virtanen, P.; Gommers, R.; Oliphant, T. E.; Haberland, M.; Reddy, T.; Cournapeau, D.; Burovski, E.; Peterson, P.; Weckesser, W.; Bright, J.; van der Walt, S. J.; Brett, M.; Wilson, J.; Millman, K. J.; Mayorov, N.; Nelson, A. R. J.; Jones, E.; Kern, R.; Larson, E.; Carey, C. J.; Polat, İ.; Feng, Y.; Moore, E. W.; VanderPlas, J.; Laxalde, D.; Perktold, J.; Cimrman, R.; Henriksen, I.; Quintero, E. A.; Harris, C. R.; Archibald, A. M.; Ribeiro, A. H.; Pedregosa, F.; van Mulbregt, P. SciPy 1.0: Fundamental Algorithms for Scientific Computing in Python. *Nat. Methods* **2020**, *17* (3), 261–272.

(10) Fabian, P.; Michel, V.; Grisel, O.; Blondel, M.; Prettenhofer, P.; Weiss, R.; Vanderplas, J.; Cournapeau, D.; Pedregosa, F.; Varoquaux, G.; Gramfort, A.; Thirion, B.; Grisel, O.; Dubourg, V.; Passos, A.; Brucher, M.; Perrot, M.; Duchesnay, É. *Scikit-Learn: Machine Learning in Python*; 2011; Vol. 12.

(11) Hunter, J. D. Matplotlib: A 2D Graphics Environment. *Comput. Sci. Eng.* **2007**, *9* (3), 90–95.

(12) Humphrey, W.; Dalke, A.; Schulten, K. VMD: Visual Molecular Dynamics. *J. Mol. Graph.* **1996**, *14* (1), 33–38.

(13) Ingólfsson, H. I.; Melo, M. N.; Van Eerden, F. J.; Arnarez, C.; Lopez, C. A.; Wassenaar, T. A.; Periole, X.; De Vries, A. H.; Tieleman, D. P.; Marrink, S. J. Lipid Organization of the Plasma Membrane. *J. Am. Chem. Soc.* **2014**, *136* (41), 14554–14559.

(14) Goose, J. E.; Sansom, M. S. P. Reduced Lateral Mobility of Lipids and Proteins in Crowded Membranes. *PLoS Comput. Biol.* **2013**, *9* (4), e1003033.

(15) Reddy, T.; Shorthouse, D.; Parton, D. L.; Jefferys, E.; Fowler, P. W.; Chavent, M.; Baaden, M.; Sansom, M. S. P. Nothing to Sneeze At: A Dynamic and Integrative Computational Model of an Influenza A Virion. *Structure* **2015**, *23* (3), 584–597.

(16) Salfer, M.; Collado, J. F.; Baumeister, W.; FernándezBusnadiego, R.; Martínez-Sánchez, A. Reliable Estimation of Membrane Curvature for Cryo-Electron Tomography. *PLoS Comput. Biol.* **2020**, *16* (8), e1007962.

(17) Lakowicz, J. R. *Principles of Fluorescence Spectroscopy*; Springer, 2006.

(18) Gendron, P.-O.; Avaltroni, F.; Wilkinson, K. J. Diffusion Coefficients of Several Rhodamine Derivatives as Determined by Pulsed Field Gradient–Nuclear Magnetic Resonance and Fluorescence Correlation Spectroscopy. *J. Fluoresc.* **2008**, *18* (6), 1093–1101.
